# Optimization of incubation conditions of *Plasmodium falciparum* antibody multiplex assays to measure IgG, IgG_1–4_, IgM and IgE using standard and customized reference pools for sero-epidemiological and vaccine studies

**DOI:** 10.1186/s12936-018-2369-3

**Published:** 2018-06-01

**Authors:** Itziar Ubillos, Alfons Jiménez, Marta Vidal, Paul W. Bowyer, Deepak Gaur, Sheetij Dutta, Benoit Gamain, Ross Coppel, Virander Chauhan, David Lanar, Chetan Chitnis, Evelina Angov, James Beeson, David Cavanagh, Joseph J. Campo, Ruth Aguilar, Carlota Dobaño

**Affiliations:** 1 ISGlobal, Hospital Clínic-Universitat de Barcelona, Carrer Rosselló 153 (CEK Building), 08036 Barcelona, Catalonia Spain; 2 CIBER Epidemiología y Salud Pública (CIBERESP), Barcelona, Spain; 3 Bacteriology Division, MHRA-NIBSC, South Mimms, Potter Bars, EN6 3QG UK; 4 Laboratory of Malaria and Vaccine Research, School of Biotechnology, Jawaharlal Nehru University, New Delhi, India; 5 Malaria Group, International Centre for Genetic Engineering and Biotechnology (ICGEB), New Delhi, India; 6 U.S. Military Malaria Vaccine Program, Walter Reed Army Institute of Research, Silver Spring, MD USA; 7 Université Sorbonne Paris Cité, Université Paris Diderot, Inserm, INTS, Unité Biologie Intégrée du Globule Rouge UMR_S1134, Laboratoire d’Excellence GR-Ex, Paris, France; 8 Infection and Immunity Program, Monash Biomedicine Discovery Institute and Department of Microbiology, Monash University, Clayton, VIC Australia; 9 Macfarlane Burnet Institute for Medical Research and Public Health, Melbourne, VIC Australia; 10 Institute of Immunology & Infection Research and Centre for Immunity, Infection & Evolution, Ashworth Laboratories, School of Biological Sciences, University of Edinburgh, King’s Buildings, Charlotte Auerbach Rd, Edinburgh, EH9 3FL UK

**Keywords:** *Plasmodium falciparum*, Quantitative suspension array technology, Multiplex, IgG, IgG1, IgG2, IgG3, IgG4 subclasses, IgM, IgE, Reference reagent, Incubation conditions, Assay performance

## Abstract

**Background:**

The quantitative suspension array technology (qSAT) is a useful platform for malaria immune marker discovery. However, a major challenge for large sero-epidemiological and malaria vaccine studies is the comparability across laboratories, which requires the access to standardized control reagents for assay optimization, to monitor performance and improve reproducibility. Here, the *Plasmodium falciparum* antibody reactivities of the newly available WHO reference reagent for anti-malaria human plasma (10/198) and of additional customized positive controls were examined with seven in-house qSAT multiplex assays measuring IgG, IgG_1–4_ subclasses, IgM and IgE against a panel of 40 antigens. The different positive controls were tested at different incubation times and temperatures (4 °C overnight, 37 °C 2 h, room temperature 1 h) to select the optimal conditions.

**Results:**

Overall, the WHO reference reagent had low IgG2, IgG4, IgM and IgE, and also low anti-CSP antibody levels, thus this reagent was enriched with plasmas from RTS,S-vaccinated volunteers to be used as standard for CSP-based vaccine studies. For the IgM assay, another customized plasma pool prepared with samples from malaria primo-infected adults with adequate IgM levels proved to be more adequate as a positive control. The range and magnitude of IgG and IgG_1–4_ responses were highest when the WHO reference reagent was incubated with antigen-coupled beads at 4 °C overnight. IgG levels measured in the negative control did not vary between incubations at 37 °C 2 h and 4 °C overnight, indicating no difference in unspecific binding.

**Conclusions:**

With this study, the immunogenicity profile of the WHO reference reagent, including seven immunoglobulin isotypes and subclasses, and more *P. falciparum* antigens, also those included in the leading RTS,S malaria vaccine, was better characterized. Overall, incubation of samples at 4 °C overnight rendered the best performance for antibody measurements against the antigens tested. Although the WHO reference reagent performed well to measure IgG to the majority of the common *P. falciparum* blood stage antigens tested, customized pools may need to be used as positive controls depending on the antigens (e.g. pre-erythrocytic proteins of low natural immunogenicity) and isotypes/subclasses (e.g. IgM) under study.

**Electronic supplementary material:**

The online version of this article (10.1186/s12936-018-2369-3) contains supplementary material, which is available to authorized users.

## Background

The identification of immune correlates of protection and risk against malaria is particularly challenging when dealing with a complex pathogen like *Plasmodium falciparum,* which has a proteome of over 5000 proteins (http://www.plasmodb.org), some of them polymorphic and/or variant. Consequently, malaria infection induces a very broad and diverse antigen-specific immunoglobulin (Ig) subtype response [1, 2]. Although the crucial role of IgG antibodies in protective malaria immunity was demonstrated long time ago [3, 4], the antigenic targets of these antibodies have not yet been identified. However, it is presumed that such IgG responses are primarily directed to antigens on the surface of the *P. falciparum* asexual blood stage (BS). Numerous immune-epidemiological surveys have reported significant associations between levels of BS-specific IgG antibodies and protection from clinical malaria [5–7]. However, most of these studies have only described the magnitude of IgG responses and little is known about their subtypes, quality and functionality. Thus, the mechanisms mediating antibody immunity are not fully elucidated.

Early in vitro studies suggested that inhibitory IgG antibodies may control *P. falciparum* growth in collaboration with monocytes through opsonic phagocytosis [8–10] or antibody-dependent cellular inhibition [11]. Collectively, studies have pointed to cytophilic IgG subclasses (IgG1 and IgG3) as the main contributors to naturally-acquired immunity, suggesting that cells bearing Fc-g receptors are involved in protective immune mechanisms [12–16]. Recent studies have also highlighted the potential importance of IgM [17, 18] or IgE [19, 20] in malaria protection or risk, respectively, but these isotypes have been much less studied in the malaria field. Further studies addressing antibody isotypes, subclasses, and their antigenic breadth are needed to define correlates in natural and in artificial immunity induced by vaccines such as the RTS,S/AS01E and those based on attenuated sporozoites.

RTS,S/AS01E is the most advanced malaria vaccine in development globally [21], however the immune surrogates of protection, the mode of action, and how vaccination affects or is affected by naturally-acquired immunity, remain unclear. A better characterization of the malaria serological profile at the Ig isotype and subclass levels could help address these questions. However, widely applicable standardized, miniaturized, multiplex, high-throughput assays, able to measure all Ig isotypes and subclasses, have been lacking.

The quantitative suspension array technology (qSAT) is an optimal platform for malaria biomarker discovery. The qSAT is a mid-high throughput platform that allows measuring multiple antigen-specific antibodies (up to 500) in small sample volumes and in one single reaction. To study the mechanisms of immunity in malaria, several in-house qSAT assays using panels of up to 15 *P. falciparum* antigens were previously developed to measure total IgG [22], IgG_1–4_, IgM and IgE [23] and factors affecting IgG assay variability evaluated (Ubillos et al., pers. comm.). However, a major challenge in the development of serological tests has been the lack of standardized positive controls [24] to allow comparability of data generated in different assays and laboratories, particularly when assessing large antigenic panels and diverse antibody isotypes/subclasses in samples of heterogeneous origin. Recently, a *P. falciparum*-specific human serum reference reagent (10/198) stable at high temperature and up to 24 months of storage has been described [25] that reduced inter-laboratory variation. This WHO standard has been characterized by ELISA to contain IgGs that recognize the circumsporozoite surface protein (CSP) and a handful of *P. falciparum* antigens from different genotypes: the merozoite surface protein (MSP)-1_19_ (K1 strain), MSP-1_42_ (3D7), MSP-2 (3D7), MSP-3 (K1), and the apical membrane antigen (AMA)-1 (3D7, FC27 and FP3). The malaria community would benefit from having wider information on antigenic recognition of this reference reagent.

In previous studies, antigen-coupled beads were incubated with samples for 1 h at room temperature [22, 23, 26, 27]. Temperature of incubation influences the antigen–antibody affinity [28, 29] and 1 h might not ensure the appropriate association/dissociation equilibrium. Hence, expanded incubation times with lower (4 °C) and higher (37 °C) temperatures could affect the assay performance.

In this study, a broader antibody reactivity profile of the WHO reference reagent and other customized positive controls was examined with seven in-house qSAT antibody assays measuring IgG, IgG_1–4_, IgM and IgE against a panel of 40 antigens, including *P. falciparum* proteins that are part of the RTS,S/AS01E vaccine. This information will be generalizable to other applications and large sero-epidemiological and vaccine studies of sporozoite and BS antigen targets, being useful for the malaria research community as a whole. In addition, different sample incubation times and temperatures (4 °C overnight, 37 °C 2 h, room temperature 1 h) were tested to select the incubation conditions rendering the optimal quantification range and higher sensitivity without increasing unspecific binding.

## Methods

### Antigens

A customized multiplex panel with 33 BS and 6 pre-erythrocytic (PE) *P. falciparum* antigens was established (Table 1). The glycan α-Gal (Gala1–3GalB1–4GlcNAc-R), detected in the surface of sporozoites, was also included, as anti-α-Gal IgM antibodies have been associated with malaria protection [30]. In addition to *P. falciparum* antigens, the hepatitis B surface antigen (HBsAg, a component of the RTS,S vaccine) was added, as the assays were intended to be used with samples from this vaccine trial. Also, bovine serum albumin (BSA) and glutathione S-transferase (GST) were added to the panel to control for background signal coming from unspecific binding to the BSA used to block the coupled beads, and to the GST present in some of the fusion proteins.

**Table 1:** Antigens included in the multiplex qSAT panel

Antigens and genotype		Life-cycle stage	Rationale	References
Pre-erythrocytic (PE)				
CelTOS		Sporozoite	Exposure to sporozoite	[31, 32]
CSP full length*		Sporozoite	Exposure to sporozoite and RTS,S specific	[30, 33]
CSP NANP repeat*	GST-fused	Sporozoite	Exposure to sporozoite and RTS,S specific	[35]
CSP C-terminus*	GST-fused	Sporozoite	Exposure to sporozoite	[36]
SSP2 or TRAP		Sporozoite	Representative of exposure to sporozoite	[34, 37]
Liver stage				
LSA-1*		Liver stage	Liver stage antigen—infected hepatocytes	[38, 39]
Blood stage (BS)				
AMA-1 3D7 (FMP2.1)*		Merozoite	Involved in erythrocyte invasion	[40, 41]
AMA-1 FVO (FMP009)		Merozoite	Involved in erythrocyte invasion	[41]
CyRPA full length		Merozoite	Involved in erythrocyte invasion	[42]
EBA-140	GST-fused	Merozoite	Involved in erythrocyte invasion	[43]
EBA-175 R2 PfF2		Merozoite	Involved in erythrocyte invasion	[44]
EBA-175 R3–5*	GST-fused	Merozoite	Involved in erythrocyte invasion	[43]
EXP-1		Merozoite	Involved in erythrocyte invasion	[45]
MSP-1 Block 2 3D7*	GST-fused	Merozoite	Involved in erythrocyte invasion	[46]
MSP-1 Block 2 hybrid	GST-fused	Merozoite	Involved in erythrocyte invasion	[47]
MSP-1 Block 2 MAD20	GST-fused	Merozoite	Involved in erythrocyte invasion	[46]
MSP-1 Block 2 PA17	GST-fused	Merozoite	Involved in erythrocyte invasion	[46]
MSP-1 Block 2 RO33	GST-fused	Merozoite	Involved in erythrocyte invasion	[46]
MSP-1 Block 2 Well	GST-fused	Merozoite	Involved in erythrocyte invasion	[46]
MSP-1_42_ 3D7*		Merozoite	Involved in erythrocyte invasion	[41, 48]
MSP-1_42_ FVO		Merozoite	Involved in erythrocyte invasion	[41, 48]
MSP-2 full length B*	GST-fused	Merozoite	Representative of exposure to BS	[49]
MSP-2 full length A*	GST-fused	Merozoite	Representative of exposure to BS	[49]
MSP-3 3C		Merozoite	Representative of exposure to BS	[49]
MSP-3 3D7*		Merozoite	Representative of exposure to BS	[50]
MSP-5		Merozoite	Representative of exposure to BS	[51, 52]
MSP-6*	GST-fused	Merozoite	Representative of exposure to BS	[53]
P41		Merozoite	Involved in erythrocyte invasion	[54]
RH1		Merozoite	Involved in erythrocyte invasion	[55]
RH2 (2030)	GST-fused	Merozoite	Involved in erythrocyte invasion	[56]
RH2 b240		Merozoite	Involved in erythrocyte invasion	[57]
RH4.2	GST-fused	Merozoite	Involved in erythrocyte invasion	[58, 59]
RH4.9*		Merozoite	Involved in erythrocyte invasion	[58, 59]
RH5		Merozoite	Involved in erythrocyte invasion	[42, 60]
PTRAMP		Merozoite	Involved in erythrocyte invasion	[61]
DBL-α		Trophozoite	Involved in cytoadherence	[62]
Pregnancy-specific				
DBL1-DBL2 VAR2CSA		Trophozoite	Associated to placental malaria exposure and representative of maternally-transferred antibodies	[63]
DBL3-DBL4 VAR2CSA*		Trophozoite		[64]
Other antigens				
HBsAg*		NA	Hepatitis B surface antigen	
α-Gal			Involved in malaria protection	[30]
Controls				
GST*		Background	Control fusion protein	
BSA*		Background	Control unspecific binding	

* Recombinant proteins used for the experimental assessment of the optimal temperature and time of samples incubation in the IgG assays. MSP-2 A corresponds to the CH150 strain and MSP-2 B to the Dd2 strain

### Coupling of antigens to microspheres

Coupling of carboxylated polystyrene microspheres was carried out as described elsewhere [26]. Briefly, MagPlex^®^ microspheres (Luminex Corp., Austin, Texas) with different spectral signatures selected for each antigen, were washed with distilled water and activated with Sulfo-NHS (*N*-hydroxysulfosuccinimide) and EDC (1-ethyl-3-[3-dimethylaminopropyl]carbodiimide hydrochloride) (Pierce, Thermo Fisher Scientific Inc., Rockford, IL), both at 50 mg/mL, in activation buffer (100 mM Monobasic Sodium Phosphate, pH 6.2). Microspheres were washed with 50 mM MES (4-morpholineethane sulfonic acid, Sigma, Tres Cantos, Spain) pH 5.0 or dPBS (Dulbecco’s Phosphate Buffered Saline, Lonza) pH 7.0 to a 10,000 beads/µL concentration, and coated with antigens at a concentration previously established in MES or PBS and incubated in a rotatory shaker overnight (ON) at 4 °C and protected from light. Microspheres were blocked with PBS-BN [PBS with 1% BSA and 0.05% sodium azide (Sigma, Tres Cantos, Spain)] and re-suspended in PBS-BN to be quantified on a Guava PCA desktop cytometer (Guava, Hayward, CA) to determine the percentage recovery after the coupling procedure. Antigen-coupled beads were validated in singleplex and multiplex by measuring IgG in serial dilutions of a positive control. Similar IgG MFI levels were obtained in singleplex and multiplex measurements, with a strong correlation for all antigens assessed (R^2^ > 0.98; p < 0.05) (Additional file 1). Coupled beads were stored multiplexed at a concentration of 1000 beads/µL/antigen at 4 °C and protected from light.

### Reference reagents and control samples

*WHO Reference Reagent for anti*-*malaria (P. falciparum) human plasma (10/198)* (referred as WHO reference reagent). A pool derived from plasma donations collected at the Blood Bank from individuals based in Kisumu, Kenya, with a history of malaria. This reference reagent presents IgG reactivity to *P. falciparum* AMA-1, MSP-1_19_, MSP-1_42_, MSP-2 and MSP-3 [25]. The reagent has been defibrinated and diluted (1:5) with deionized sterile water and filled into 1 mL/ampoules. Each ampoule has been lyophilized comprising a freeze-dried residue of diluted human plasma.

*RTS,S vaccine positive control* (referred as WHO-CSP pool). An RTS,S pool prepared with plasmas from 10 Mozambican children vaccinated with RTS,S/AS02 with known high IgG titres to CSP at peak response [65] was added to the WHO reference reagent (1:50 WHO reference reagent + 1:100 RTS,S pool), creating a CSP and HBsAg antibody enriched WHO reference reagent.

*Malaria primo*-*infected plasma pool* (referred as IgM pool). A customized pool prepared with plasmas from 20 malaria naïve European adults with known high anti-malaria IgM levels after being experimentally infected with *P. falciparum* in a controlled human malaria infection (CHMI) trial [66]. To prepare the pool, we first selected the time point that elicited the highest IgM breadth of response to a panel of 20 BS and 1 PE antigens from the CHMI trials conducted in Barcelona (day 35) and Tübingen (day 84). Ten individuals from each trial with the highest IgM breadth of response were selected and pooled.

*Negative control*. A pool of plasma samples from 20 Spanish malaria-naïve individuals.

*RTS,S samples.* Three samples from individuals participating in the RTS,S malaria vaccine phase 2b trial conducted in Mozambique [65] were randomly selected. High, medium and low responders were defined by tertiles.

### qSAT assay and incubation conditions tested

IgG, IgG_1–4_ subclasses, IgM and IgE levels were measured in the WHO reference reagent and other customized pools against multiplexed *P. falciparum* antigens using the xMAP™ technology (Luminex Corp., Austin, Texas). Fifty microliter of multiplexed antigen-coupled beads were added to a 96-well μClear^®^ flat bottom plate (Greiner Bio-One, Frickenhausen, Germany) at 1000 beads/analyte/well. To assess the optimal temperature and duration of sample incubation for IgG and IgG_1–4_ assays, 50 µL of WHO reference reagent at 11 serial dilutions (1:3, starting at 1/150) and the negative control at 4 serial dilutions (1:2, starting at 1:50) were incubated against a panel of 14 *P. falciparum* antigen-coated beads in a 96-well plate (Table 1). Plates were incubated in a rotatory shaker at 600 rpm and protected from light under three conditions: (i) 37 °C for 2 h; (ii) 4 °C ON and (iii) room temperature (RT) for 1 h. For the IgM assay, 50 µL of the WHO reference reagent or the IgM pool were assayed in 15 serial dilutions (1:3, starting at 1/50) against a panel of 40 *P. falciparum* antigens plus HBsAg (Table 1). Plates were incubated at two different conditions: 37 °C for 2 h and 4 °C ON. IgE levels in the WHO reference reagent assayed at 8 serial dilutions (1:2, starting at 1/10) were also measured under two different incubation conditions: 37 °C for 2 h and 4 °C ON. Finally, using the WHO-CSP pool, 23 standard curves for IgG, IgG1, IgG3 and IgM were constructed; and 12 standard curves for IgG2 and IgG4 all of 18 serial dilutions (1:2, starting at 1:50). The standard curves were incubated at 4 °C ON against a total of 40 antigens (Table 1). Beads coupled with BSA and GST were included in the panel as background controls to assess unspecific binding to BSA and GST. After the incubation, plates were washed with PBS-0.05% Tween 20 buffer using a manual magnetic washer platform (Bio-Rad, Hercules, CA, USA). Secondary antibodies were added as previously described [23]. Briefly, biotinylated anti-human IgG at 1:2500 (Sigma B1140, polyclonal), anti-human IgM at 1:1000 (Sigma B1265, polyclonal) anti-human IgG3 at 1:1000 (Sigma B3523, clone HP-6050), and anti-human IgG1 at 1:4000 (Abcam ab99775, clone 4E3). For the IgG2, IgG4 and IgE assays, the secondary antibodies were unconjugated to biotin: mouse anti-human IgG2 at 1:500 (Thermo Fisher MA1-34755, clone HP6014), mouse anti-human IgG4 at 1:8000 (Thermo Fisher MA1-80332, clone HP6025), and mouse anti-human IgE at 1:500 (Abcam ab99834, clone HP6029). All secondary antibodies were incubated 60 min at RT and washed. In IgG2, IgG4 and IgE assays, a tertiary biotinylated goat anti-mouse IgG (Sigma B7401, polyclonal) was added and incubated 60 min at RT. Plates were washed as before and streptavidin-R-phycoerythrin at 1:1000 (Sigma, Tres Cantos, Spain) was added to all wells and incubated 30 min at RT. Plates were washed and beads re-suspended in 100 μL/well of PBS-BN, protected from light and stored ON at 4 °C to be read the next day. Plates were read using the Luminex xMAP^®^ 100/200 analyser (Luminex Corp., Austin, Texas) and at least 50 microspheres per analyte were acquired per well. Results are expressed as Median Fluorescence Intensity (MFI).

RTS,S-induced antibodies were measured in 3 samples from RTS,S-vaccinated children with known high, medium and low responses, together with serial dilutions of the WHO reference reagent or the IgM pool (1:3 starting at 1:50 for IgG, IgG_1–4_ and IgM, and 1:2 starting at 1:10 for IgE) and incubated at 4 °C ON. Samples were assayed in 4 serial dilutions (1:10) starting at 1:500 for IgG, in 3 serial dilutions (1:10) starting at 1:100 for IgM and IgG1, and in 2 serial dilutions (1:10) starting at 1:50 for IgG2 and IgG4. Samples were not assayed for IgG3 or IgE.

### Statistical analyses

To stabilize the variance, the analysis was done on log_10_-transformed values of the MFI measurements. The correlation and reliability between the different sample incubation conditions for the IgG and IgG_1–4_ subclasses measured in the positive control, the negative control and the blanks were evaluated. After the Shapiro–Wilk normality test was applied, differences between conditions were assessed by Kruskal–Wallis test with posthoc Tukey test. Reliability was assessed by the interclass correlation coefficient (ICC) [67]. Titration curves of antibody concentrations vs. MFIs per antigen were fitted using a five-parameter (5PL), a 4PL or an exponential logistic equation depending on the best yield, following the formula MFI = Emax + ((Emin − Emax)/((1 + ((Conc/EC_50_)^Hill))^Asym)), where EC_50_ is the half maximal effective concentration, Emin is the minimum response, Emax is the maximum response, Asym is the asymmetry factor and Hill is the slope factor [68], using the drLumi package [69]. We calculated the coefficient of variation (CV) of Emin, Emax and used the goodness of fit model to assess the fitting of the curves. All analyses were done using R version 3.4.1.

## Results

### Total IgG, IgG_1–4_, IgM and IgE responses against RTS,S antigens in the WHO reference reagent compared to those measured in sera from RTS,S-vaccinated children

**Fig. 1:**
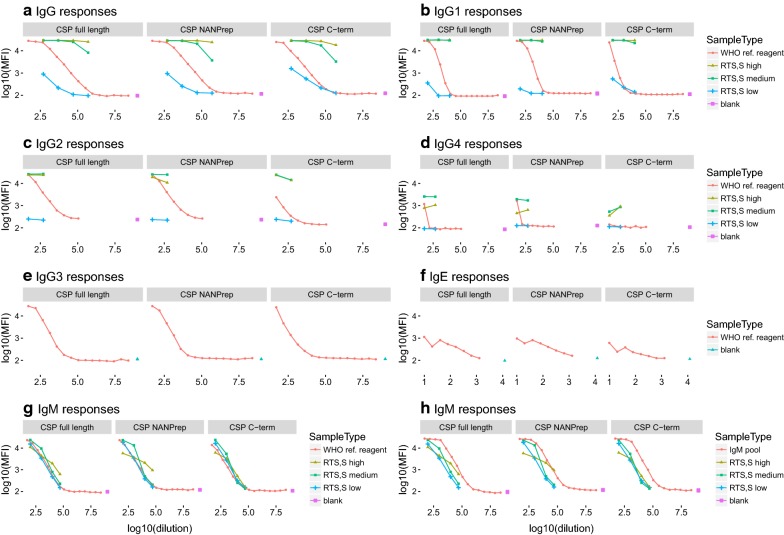
RTS,S-specific responses measured in the WHO reference reagent, IgM pool and samples from RTS,S-vaccinated children. The 3 samples from RTS,S vaccinated children were of high, medium and low CSP IgG titres. **a**–**g** IgG, IgG_1–4_, IgM and IgE levels to RTS,S-specific antigens measured in the WHO reference reagent; IgG, IgG 1, IgG2 and IgG4 also measured in RTS,S-vaccinated children; **h** IgM levels to RTS,S-specific antigens measured in the IgM pool vs. RTS,S-vaccinated children. The plots represent the levels of antibodies measured in serial dilutions of the positive pools (1:3 starting at 1:50 for IgG, IgG_1–4_ and IgM; and 1:2 starting at 1:10 for IgE), and the RTS,S vaccinees samples (1:10 starting at 1:500 for IgG, 1:100 for IgM, 1:50 for IgG_1–4_; and 1:2 starting at 1:10 for IgE). Isolated dots represent the levels measured in the technical blanks

To assess the suitability of the WHO reference reagent as a positive control to capture all responses in the context of RTS,S vaccine studies, levels of IgG, IgG_1–4_, IgM and IgE against the RTS,S-specific antigens (CSP full length, CSP NANP repeat and CSP C-terminus) were measured and compared to levels in sera from RTS,S-vaccinated children from a phase 2b trial with known IgG CSP titres [65] (Fig. 1). The WHO reference reagent and RTS,S-vaccinees antibody responses to the whole antigenic panel (Table 1) are shown in the Additional file 2. The IgM pool and RTS,S-vaccinees IgM levels were also compared (Fig. 1h and Additional file 2). The WHO reference reagent presented lower IgG, IgG1, IgG2, IgG4, and IgM levels to RTS,S antigens than samples from RTS,S-vaccinated children who had high CSP responses (Fig. 1a–d, g). Comparisons of IgG3 and IgE levels were not possible because these data were not available for RTS,S samples. The IgM pool presented higher IgM levels to RTS,S antigens than the WHO reference reagent and the RTS,S samples. Consequently, we decided to prepare a customized positive control for the RTS,S immunological studies, containing 1:50 of the WHO reference reagent plus 1:100 of pooled plasma from RTS,S-vaccinated children with high CSP titres (WHO-CSP pool). IgG responses were compared between the WHO-CSP pool and the WHO reference reagent (Additional file 3). In addition, the EC_50_ ratio between positive controls (EC_50_ WHO reference reagent/EC_50_ WHO-CSP) was calculated for RTS,S-specific antigens as a proxy measure of relative potency of the WHO-CSP pool to the WHO reference reagent (Additional file 4). The EC_50_ ratio for the 3 CSP antigens was between 0.44 and 0.58 for IgG, IgG1 and IgG3, and close to 1 for IgG2 against CSP full length.

Fifteen proteins in the multiplex panel were GST-fused (Table 1). The WHO-CSP pool was reactive to GST because the sera from RTS,S vaccinees 1 month after primary vaccination had antibodies that cross-reacted with GST. However, even if the samples contained equal or higher levels of antibodies to GST, this did not impede to accurately measure anti-malarial antibodies to the GST fusion proteins, as shown in correlation analyses of GST vs. GST fusion proteins in plasmas from RTS,S vaccinees (Additional file 5).

### Levels of total IgG, IgG_1–4_ and IgM against multiple *P. falciparum* antigens plus HBsAg measured in the WHO-CSP pool

**Fig. 2:**
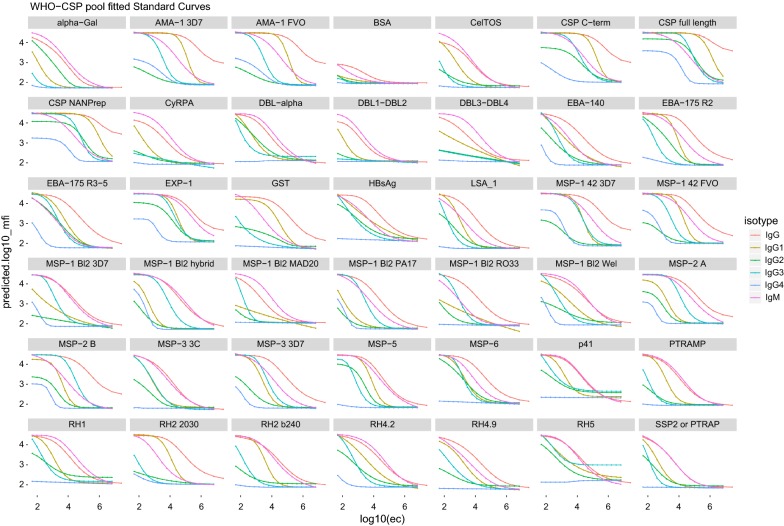
IgG, IgG_1–4_ and IgM fitted curves using the WHO-CSP pool to the 40-antigen multiplex panel incubating at 4 °C ON. Lines and dots represent predicted levels from 5PL, 4PL or exponential regression equations from 23 titration curves for IgG, IgG1, IgG3 and IgM; and 12 curves for IgG2 and IgG4. Titration curves contained 18 serial dilutions (1:2) starting at 1/50 of the WHO-CSP pool to a panel of 39 *P. falciparum* antigens plus HBsAg, α-Gal, BSA and GST

The WHO-CSP pool was used to generate IgG, IgG_1–4_ and IgM titration curves incubating at 4 °C ON in the context of an RTS,S immunology study. The level of response was antigen-dependent; the most immunogenic proteins (AMA-1 3D7 and FVO, MSP-1_42_ 3D7 and FVO) gave saturated signals even at the 1:6.5 × 10^6^ dilution (Fig. 2).

**Fig. 3:**
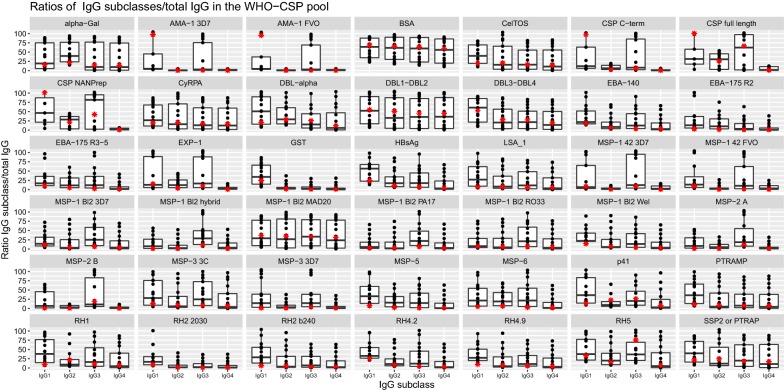
Boxplots of ratios of IgG_1–4_ subclasses to total IgG measured in the WHO-CSP pool. Ratios are composed with the median of the 23 titration curves for IgG, IgG1 and IgG3 and 12 curves for IgG2 and IgG4, for each dilution point. Boxes show medians and interquartile ranges. The red star corresponds to the ratio of the median of each dilution of IgG subclass to the median of each dilution of total IgG

To further characterize the IgG subclass composition of the WHO-CSP pool, the ratios of IgG_1–4_ subclasses to total IgG [MFI IgG subclass at dilution (i)/MFI total IgG at dilution (i) × 100] were measured (Fig. 3). The predominance of IgG subclasses also varied depending on the antigen. For example, IgG1 responses were dominant for HBsAg, LSA-1, MSP-5, P41, RH1, RH2, PTRAMP, RH4.2, RH4.9 and SSP2, whereas MSP-2 full length, MSP-1 block 2 and RH4 induced mainly IgG3. IgG subclass responses to AMA-1 (3D7 and FVO), CSP (C-terminus and NANP repeat), EXP-1, MSP-1_42_ (3D7 and FVO), MSP-3 and RH5 were dominated by IgG1 and IgG3.

### Optimal temperature and time of incubation to measure IgGs against *P. falciparum* antigens using the WHO reference reagent

**Fig. 4:**
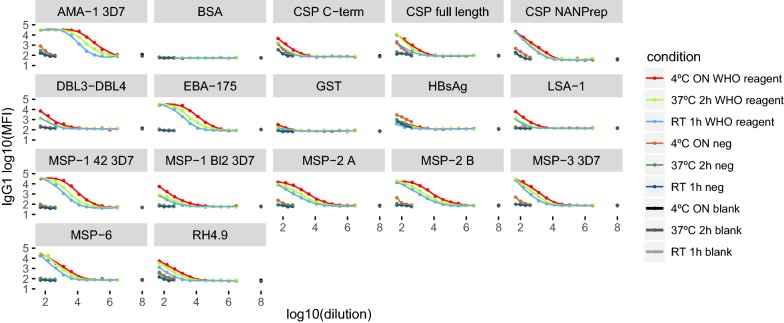
Levels of IgG1 measured to 15 antigens in the WHO reference reagent compared to negative control and blanks under three different incubation conditions. Curve plots of the antigen-specific IgG1 levels measured in serial dilutions of the WHO reference reagent, negative control and blanks at three different incubation conditions: 37 °C 2 h, 4 °C overnight (4 °C ON) and room temperature 1 h (RT 1 h). “neg” means negative control

To assess the optimal temperature and time of incubation for the measurement of IgG and IgG_1–4_ subclasses, the assay performance of the WHO reference reagent against a panel of 14 *P. falciparum* antigens (Table 1) under three different incubation conditions (4 °C ON, 37 °C 2 h and RT 1 h) was compared. IgG and IgG_1–4_ assays varied depending on the incubation procedure, with the largest difference between 4 °C ON and RT 1 h (p < 0.001) for IgG. No differences were found between these two incubation conditions for IgG2, IgG3 and IgG4. Differences between 4 °C ON and 37 °C 2 h were only observed for IgG (p = 0.026). IgG and IgG_1–4_ levels against BSA and blanks were not affected by the incubation conditions. The MFI levels of IgG and IgG_1–4_ measured in the negative control only varied when comparing 4 °C ON vs. RT 1 h (p < 0.001) for some of the antigens. Figure 4 shows examples of the results for IgG1, and the complete data set is in the Additional file 6. The incubation at 4 °C ON, on average, showed the highest MFIs in the first dilution, except for IgG4 and reached blank levels at the lowest dilution (Fig. 4 and Additional file 6). Negative control MFI levels were also higher at 4 °C ON compared to other conditions, however the difference with the WHO reference reagent at same dilution was high enough to establish a positivity threshold (Fig. 4).

Correlations between incubation conditions for IgG and IgG_1–4_ subclasses measured against all antigens in the WHO reference reagent and negative control showed a r^2^ > 0.93 for all IgG and IgG_1–4_ subclasses. The ICCs between incubation conditions for IgG and IgG_1–4_ measured in the WHO reference reagent showed overall good reliability, being 0.91 (0.89–0.93) for IgG3, 0.88 (0.87–0.89) for IgG1, 0.83 (0.79–0.86) for total IgG, 0.79 (0.74–0.83) for IgG2 and 0.63 (0.53–0.72) for IgG4. However, as seen in Fig. 4 and Additional file 6, ICCs in the negative control were of lower reliability, being of 0.85 (0.78–0.9) for IgG4, 0.74 (0.64–0.82) for IgG2, 0.38 (0.23–0.53) for total IgG, 0.39 (0.23–0.54) for IgG1 and 0.11 (− 0.03–0.14) for IgG3. Blank levels were similar between incubation conditions (Fig. 4 and Additional file 6). Taking together these results, we chose the incubation at 4 °C ON as the optimal for the IgG assays.

### Optimal temperature and time of incubation to measure IgM and IgE against *P. falciparum* antigens using the WHO reference reagent and an IgM customized pool

**Fig. 5:**
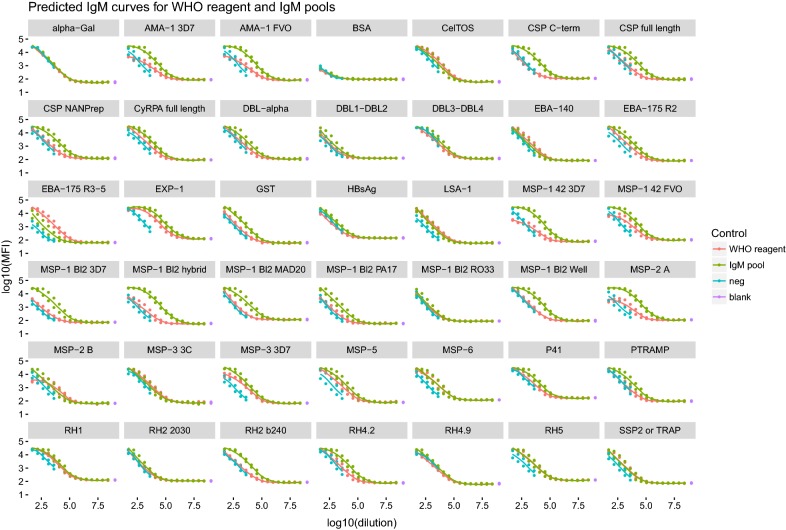
Fitted IgM curves to the 40-multiplex panel in the WHO reference reagent and the IgM pool compared to negative control and blanks under two different incubation conditions. Curves from 4PL or 5PL logistic model equation comparing IgM levels measured in the WHO reference reagent, the IgM pool, the negative control and the blanks. Isolated dots in purple represent the IgM levels measured in the technical blanks

Incubation conditions to measure IgM and IgE responses against a panel of 38 *P. falciparum* antigens plus HBsAg, α-Gal, BSA and GST (Table 1) were tested using the WHO reference reagent and an alternative IgM pool. The IgM pool gave higher IgM responses and of higher range compared to those obtained with the WHO reference reagent for most of the antigens, especially AMA-1s, MSP-1s and CSPs (Fig. 5). Incubation of the IgM pool at 4 °C ON showed higher responses compared to incubation at 37 °C 2 h (Additional file 7A), with 80% of the antigens studied (35/43) presenting a higher EC_50_ (i.e. AMA-1 3D7 EC_50_ 4 °C ON 3.64 ± 0.66 and EC_50_ 37 °C 2 h 2.62 ± 0.96). The IgM responses of the negative control measured at first dilution were higher than those of IgG and IgG subclasses, but levels dropped quickly after the first dilution. Overall, IgM pool responses showed higher difference to the negative control than those obtained with the WHO reference reagent (Fig. 5). Similar differences in IgM responses between incubation conditions were obtained with the WHO reference reagent, measuring higher levels when incubating at 4 °C ON than at 37 °C 2 h (Additional file 7B). IgM technical blanks were not affected by incubation conditions (Additional file 7A, B). Correlations for IgM responses between incubation conditions were r^2^ = 0.96 for both WHO reference reagent and IgM pool. For the IgE assay, there were no differences between incubation conditions (Additional file 7C).

The ICCs between antibody responses measured in the two incubation conditions with the WHO reference reagent were 0.92 (0.91–0.93) for IgM and 0.82 (0.79–0.85) for IgE; and the ICC between conditions for the IgM assay using the IgM pool was 0.91 (0.9–0.92). However, IgM responses of negative controls showed moderate reliability between incubation conditions, having an ICC of 0.66 (0.57–0.73).

When comparing antibody levels measured in the WHO reference reagent vs. the IgM pool, there was moderate reliability, with ICC of 0.65 (0.61–0.769) at 4 °C ON, and 0.66 (0.61–0.7) at 37 °C 2 h, meaning that there was 35% of variability between reference pools. Considering the strong correlation and reliability of the two incubation conditions, but the higher IgM levels and MFI ranges obtained at 4 °C ON, this incubation was also chosen for the IgM assay.

## Discussion

A major challenge in large malaria sero-epidemiological and vaccine studies is to have access to consistent and unlimited control reagents that provide assay quality control and facilitate data consolidation. A universal malaria reference pool would be ideal to monitor performance of serological assays, improve inter-laboratory reproducibility, make data from different studies comparable, and potentially give quantitative antibody measures. In this study, information was provided on the expanded antibody reactivity profile of the commercially available WHO reference reagent for anti-malaria (*P. falciparum*) human plasma (10/198) [25] and other customized positive controls by using seven in-house qSAT multiplex antibody assays to measure IgG, IgG_1–4_, IgM and IgE against a panel of 40 antigens, including *P. falciparum* proteins that are part of the RTS,S/AS01E vaccine. In addition, different sample incubation times and temperatures (4 °C ON, 37 °C 2 h, RT 1 h) were tested for the qSAT assays to select the incubation conditions rendering the optimal quantification range and higher sensitivity without increasing unspecific binding. Data generated in this study will be useful for clinical malaria studies involving assessment of naturally-acquired immune responses as well as immunogenicity evaluation of CSP-based vaccine candidates.

The estimation of malaria antibody concentration in multiplex assays is increasingly difficult. There are not appropriate standards or reference sera available that react strongly to complex antigen panels. Antibody concentrations have been previously estimated using an anti-human IgG curve [22, 23, 26, 27]. However, the binding system and the affinity of the anti-human IgG curve differ from that of antibodies in samples or positive controls. Thus, different assay conditions give different slopes and curve parameters that could result in large deviations of concentration estimates. Thus, it has been recently reported that MFI responses measured independently from a standard curve might reflect actual variation, while estimated concentration values are dictated by the precision of the standard curve [70]. As an alternative, the use of long positive control curves provide upper and lower asymptotes for most antigens, and allow establishing the linear quantification ranges, representing the optimal range to capture the breadth of antibody response in individual samples. However, a reference human serum pool with known levels of anti-*P. falciparum* antibody concentrations is highly desirable for the malaria community. The challenge remains in sourcing adequate serum/plasma pools that cover all antigens as panels become larger and more complex.

To test the immuneprofile of the WHO reference reagent, antigen and isotype/subclass-specific curves constructed with serial dilutions of the reagent were fitted in non-linear equations, establishing the linear quantification ranges. Generation of curves with optimal linear quantification ranges is important to allow selecting the optimal dilution of test samples (lying on the linear range). In addition, the parameters of the curve may be used for the quality control of the assay. The WHO reference reagent is composed of samples from hyper-immune individuals from a malaria endemic region [25], predominantly having anti-*P. falciparum* IgG1 and IgG3 antibodies, rather than IgG2 and IgG4, reflecting the naturally-acquired antibody patterns. Thus, for most antigens, this pool is of restricted use to produce standard curves for IgG2, IgG4 or IgE antibodies, and this remains a limitation. Similarly, the WHO reference reagent might not be optimal for IgM measurements, particularly if high responses are expected in test samples. For this reason, a customized IgM pool with plasmas from naïve individuals experimentally challenged with *P. falciparum* at a time point when IgM predominated over IgG was prepared. This IgM pool proved to be very adequate for the generation of IgM titration curves in the study. Thus, as the WHO reference reagent has been established to measure IgGs, a reference standard to measure IgM responses would still be lacking. Similarly, IgG2, IgG4 and IgE specific reference standards would improve the reproducibility of the malaria-based immune assays.

This study also aimed to assess the usefulness of the WHO reference reagent as a positive control to generate titration curves in the context of RTS,S immunology studies. For this reason, samples from RTS,S vaccinated children with diverse CSP and HBsAg IgG titres were assayed together with the WHO reference reagent for comparison. It is important to test samples at several dilutions to maximize the assay sensitivity, but keeping to the minimum for cost-effectiveness, which is key in large sero-epidemiological studies. For this reason RTS,S samples were assayed at 4 dilutions for IgG, 3 dilutions for IgM and IgG1, and 2 dilutions for IgG2 and IgG4. Samples from RTS,S vaccinated children had significantly higher CSP antibodies than individuals naturally-exposed to *P. falciparum* sporozoites. Consequently, the WHO reference reagent could only be used to measure RTS,S-specific responses if a relative potency between the WHO reference reagent and the vaccinees samples was calculated [71]. Alternatively, data showed that the WHO reference reagent enriched with pooled sera from RTS,S-vaccinated children (WHO-CSP pool) [65] was adequate to capture all antibody responses, including the very high anti-CSP IgG levels in vaccinated children. To conserve the full reactivity of the WHO reference reagent to BS antigens, the WHO-CSP pool was constructed by adding half concentration of pooled plasmas from RTS,S vaccinated children (1:50 WHO reference reagent and 1:100 plasma from RTS,S vaccinees), ensuring that RTS,S specific antibodies were increased without diluting other anti-*P. falciparum* antibodies. A proxy measure of relative potency of the WHO-CSP pool vs. the WHO reference reagent was estimated with EC_50_. However, in 4PL and 5PL analysis, the dose–response is not the same over the entire tested concentration range, and the response changes relative to the concentration only in the middle part of the curves. Typically, these comparisons are made at the EC_50_, however, these calculations are only valid under limited conditions. For instance, the dose–response curve would need to have a common slope, and the maximum achievable response should be identical [72]. Unfortunately, these conditions are not met for the curves of most of the tested antigens and IgG subclasses. Similarly to CSP, it would be desirable to increase the WHO reference reagent reactivity to other *P. falciparum* PE antigens that are also vaccine candidates like SSP2/TRAP, LSA-1 or CelTOS. Additionally, a second generation of the WHO reference reagent against other *Plasmodium* species would be an advantage for other malaria immune studies in areas with *P. vivax* co-infections.

The WHO-CSP pool presented GST reactivity, mainly coming from the RTS,S samples, which poses the question of whether the GST signal could be interfering with the responses to the GST-fused proteins. However, correlation analysis showed that the antibody response to GST was not associated to the antibody response against the GST-fused protein and, therefore, that responses were independent. For example, CSP-specific antibodies detected upon vaccination were very high and not interfered by anti-GST antibodies when using CSP GST fusion proteins as capture antigens. Because of these observations, the GST values were not subtracted during data pre-processing, and it was concluded that GST reactivity was not a major part of the antibody signal to the *P. falciparum* portion of the fused proteins. Nevertheless, the GST reactivity with CSP pools remains an unsolved limitation that will be addressed in future studies upon the application of the assays to the analysis of samples from RTS,S vaccinated volunteers using GST fusion proteins, e.g. by testing the blocking of the reactivity with soluble GST.

This first WHO reference reagent contains an arbitrary unitage of 100 Units per ampoule, however the concentrations of antibodies (IgG, IgG_1–4_, IgM, IgE) specific to antigens such as those tested here remain unknown. Thus, it has been suggested to the WHO Expert Committee on Biological Standardization to assess the specific antibody concentrations in this reagent to allow absolute quantifications in future studies.

In a qSAT assay, temperature of incubation influences the reversible antigen–antibody kinetics by altering the constant association/dissociation equilibrium [29], which can impact assay sensitivity [73]. Raising the incubation temperature from 5 to 37 °C decreases the affinity of antigen–antibody complexes by decreasing the stability of the docking complex [28, 74]. The conditions previously used in our laboratory for incubation of samples with antigen-coupled beads were 1 h and RT [22, 23, 26, 27]. For this study, it was hypothesized that incubating samples for 1 h might not ensure the appropriate association/dissociation equilibrium. For this reason, expanded incubation times were tested and lower (4 °C) and higher (37 °C) temperatures were explored. Higher IgG and IgG_1–4_ levels were detected when the WHO reference reagent was incubated ON at 4 °C compared to 2 h at 37 °C or 1 h at RT. The ON incubation at 4 °C increased the IgG levels detected at high concentrations of the WHO reference reagent, but also the negative control. Yet, the difference between the WHO reference reagent and the negative control was large enough to establish a positive threshold. Different incubation conditions showed small differences for the WHO reference reagent performance, but larger differences for the negative control, indicating more variability at very low IgG concentrations. The unspecific binding of IgGs to BSA-coupled beads or the background signal in the technical blanks was not affected by the incubation conditions, suggesting that the specificity of the IgG binding was not affected by incubation duration or temperature. For all these reasons, 4 °C ON was the incubation condition chosen for the anti-*P. falciparum* IgG and IgG_1–4_ profiling of the WHO reference reagent and the WHO-CSP pool.

The optimal incubation condition for the IgM assay was assessed using the WHO reference reagent and the IgM pool. IgM levels were higher when incubating at 4 °C ON, although no significant differences were detected between incubating at 4 °C ON or 37 °C 2 h. Similarly to IgG and IgG_1–4_ subclasses, IgM levels to BSA and blanks were low and not affected by the incubation condition. Based in these observations, 4 °C ON was also the incubation condition chosen for the IgM assay.

The main limitation of the IgM assay was the high reactivity of the negative control, also affected by the duration and temperature of incubation. IgMs are the first class of antibodies produced during a primary immune response. They are generated in the absence of apparent stimulation by specific antigens [75], and are thought to aid in the neutralization of pathogens prior to the development of high affinity, antigen-specific antibodies [76]. Natural IgMs tend to have rather low antigen-binding affinities, compensated (to some extent) by their pentameric nature. Thus, IgM is a highly polyreactive antibody [28] and cross-reactivity of IgMs with antigens from other pathogens to which they have been exposed, or even pathogens that have not yet been “seen” by the host immune system [77, 78], could account for the high reactivity observed in the negative control. Additional tests are currently being performed to improve the specificity of the IgM qSAT assay.

## Conclusion

This study served to expand the characterization of the immunogenicity profile of the WHO reference reagent, including multiple Ig isotypes/subclasses, and significantly more *P. falciparum* antigens, including CSP. The study also served to establish the optimal sample incubation condition for seven qSAT assays (4 °C ON). Some of the limitations of the WHO reference reagent were circumvented by preparing in-house or adapted pools to quantify high anti-CSP IgG and IgM responses. Information generated here is applicable to other malaria sero-epidemiological studies of PE and BS vaccine candidates, and thus valuable for the malaria research community.

## Additional files

**Additional file 1.** Correlations of antigen-specific IgG levels (log_10_ MFI) between singleplex and multiplex coupled-beads measured in serial dilutions of a positive control pool. The positive pool was composed of plasmas from Mozambican adults with life-long exposure to malaria. The panel contained 26 antigens. The correlation coefficients (r^2^) are indicated, and the blue line corresponds to the linear fit.

**Additional file 2.** Comparison of the WHO reference reagent, IgM pool and RTS,S samples responses to the 40-antigen multiplex panel incubating at 4 °C ON. IgG, IgG_1–4_ subclasses, IgM and IgE were measured in the respective pools and samples. The plots represent the levels of antibodies measured in serial dilutions of the positive pools (1:3 starting at 1:50 for IgG, IgG_1–4_ and IgM; and 1:2 starting at 1:10 for IgE), and the RTS,S samples (1:10 starting at 1:500 for IgG, 1:100 for IgM, 1:50 for IgG_1–4_; and 1:2 starting at 1:10 for IgE). Data on IgG3 and IgE levels measured in RTS,S vaccinees were not available. Isolated dots represent the levels measured in the technical blanks.

**Additional file 3.** Comparison of the IgG and IgG_1–4_ predicted curves between the WHO reference reagent and the WHO-CSP pool incubating at 4 °C ON. IgG and IgG_1–4_ predicted curves from a non-linear equation were measured against a 23-multiplex panel. Isolated dots represent the levels measured in the technical blanks.

**Additional file 4.** IgG and IgG_1–4_ 50% effective concentrations (EC_50_) to RTS,S-specific antigens measured in the WHO reference reagent and the WHO-CSP pool, and EC_50_ ratios between pools. The functions used to fit the standard curves were 4PL (SSl4) or exponential (SSexp) equations.

**Additional file 5.** Correlations between GST vs. antigens included in the RTS,S vaccine, and GST vs. non-RTS,S antigens in plasmas from RTS,S-vaccinated children. Scatterplots with levels of IgG (log_10_MFI) to GST alone in the X-axis and to GST-fused proteins (orange) or proteins not fused to GST (green) in the Y-axis. Linear regression lines with 95% confidence intervals (in grey) and Spearman correlation coefficients (r^2^) for each antigen. Correlations between IgGs to RTS,S proteins and GST were high but similar between GST-fused (CSP NANP & C-terminus) and non GST-fused proteins (CSP full length and HBsAg). Antibody levels against the GST-fused CSPs (Y-axis value) were higher than to the GST alone (X-axis value). IgG levels to GST fusion proteins representing non-RTS,S antigens (e.g. EBA-175, MSP-2) were not correlated with IgG levels to GST alone. There were low antibody responses to these antigens while there was a higher signal to the GST alone. Overall, the patterns of correlations were similar between GST-fused and non-GST fused proteins. Responses to GST and to GST fusion proteins appeared to be independent.

**Additional file 6.** Levels of IgG and IgG_2-4_ to 15 antigens measured in the WHO reference reagent compared to negative control and blanks under three different incubation conditions. Curve plots of the antigen-specific antibody levels measured in serial dilutions of the WHO reference reagent, negative control and blanks at three different incubation conditions: 37 °C 2 h (37 °C 2 h), 4 °C overnight (4 °C ON) and room temperature for 1 h (RT 1 h). “neg” means negative control.

**Additional file 7.** Levels of IgM and IgE measured to the 40-multiplex panel in the WHO reference reagent and IgM pool compared to negative control and blanks under two different incubation conditions. Incubation conditions compared are: 4 °C (4 °C ON) vs 2 h at 37 °C (37 °C 2 h). A) Predicted 5PL curves of IgM levels in the IgM pool. B) Predicted 5PL curves of IgM levels in the WHO reference reagent. C) IgE levels in the WHO reference reagent.
