# Supplementary figures and images for: Optimization of incubation conditions of Plasmodium falciparum antibody multiplex assays to measure IgG, IgG1–4, IgM and IgE using standard and customized reference pools for sero-epidemiological and vaccine studies
Source: Malar J. 2018 Jun 1;17:219. doi: 10.1186/s12936-018-2369-3 (PMC5984756; doi:10.1186/s12936-018-2369-3)

IgG MFI levels singleplex vs. multiplex correlations

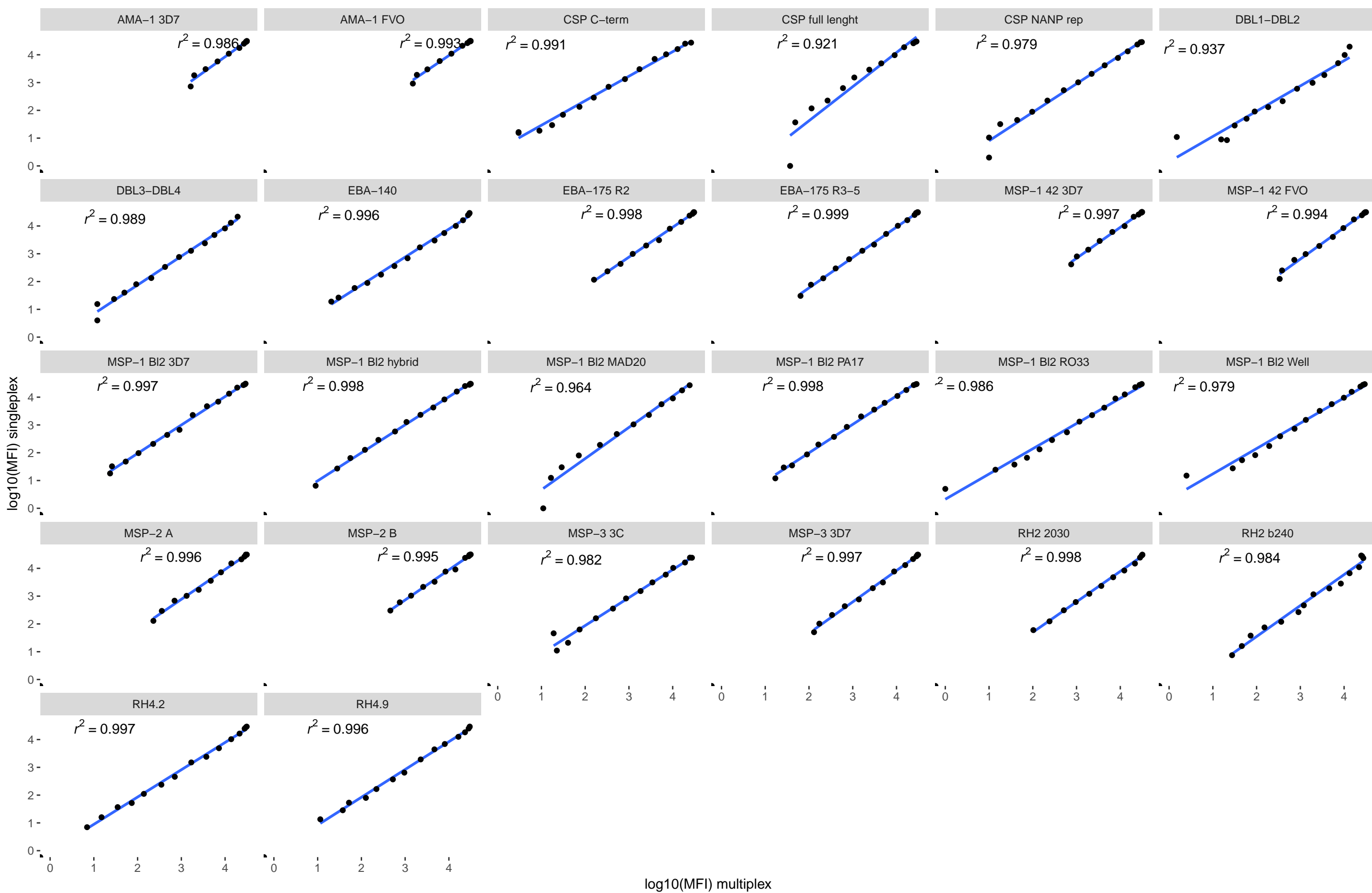

Supplement: Supplementary file 1 — Additional file 1. Correlations of antigen-specific IgG levels (log10 MFI) between singleplex and multiplex coupled-beads measured in serial dilutions of a positive control pool. The positive pool was composed of plasmas from Mozambican adults with life-long exposure to malaria. The panel contained 26 antigens. The correlation coefficients (r2) are indicated, and the blue line corresponds to the linear fit. [file 12936_2018_2369_MOESM1_ESM.pdf]

# IgG responses

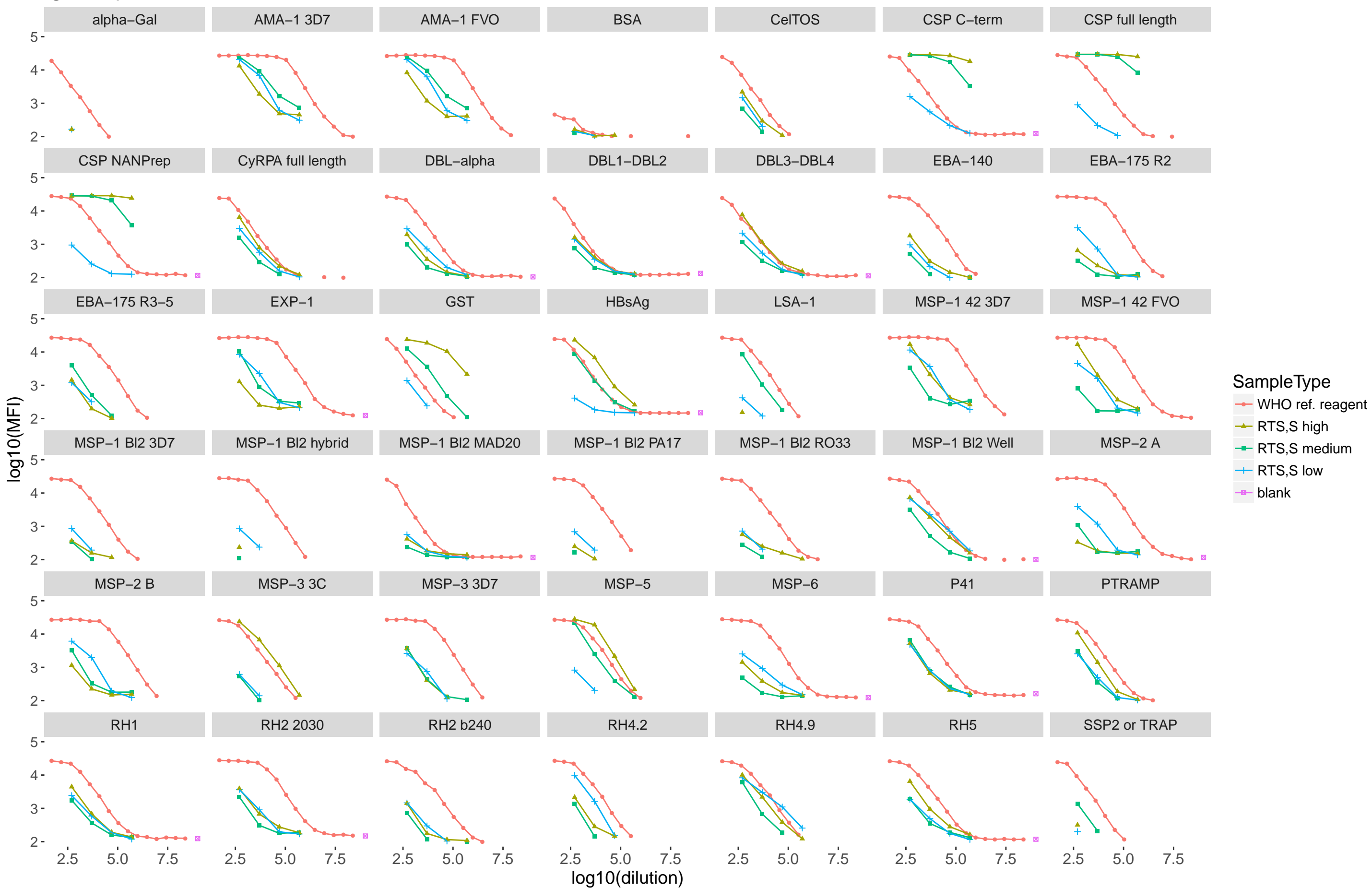

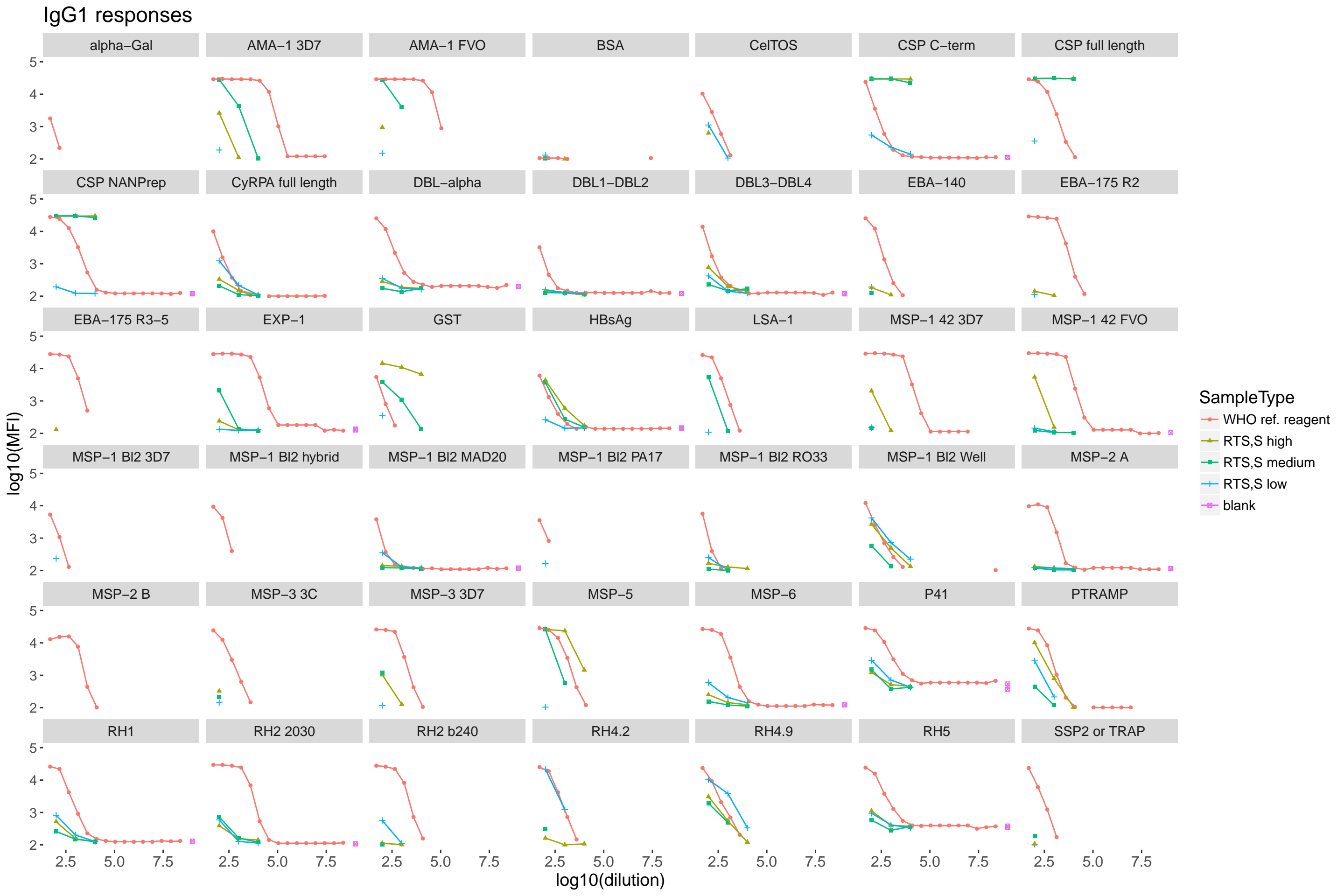

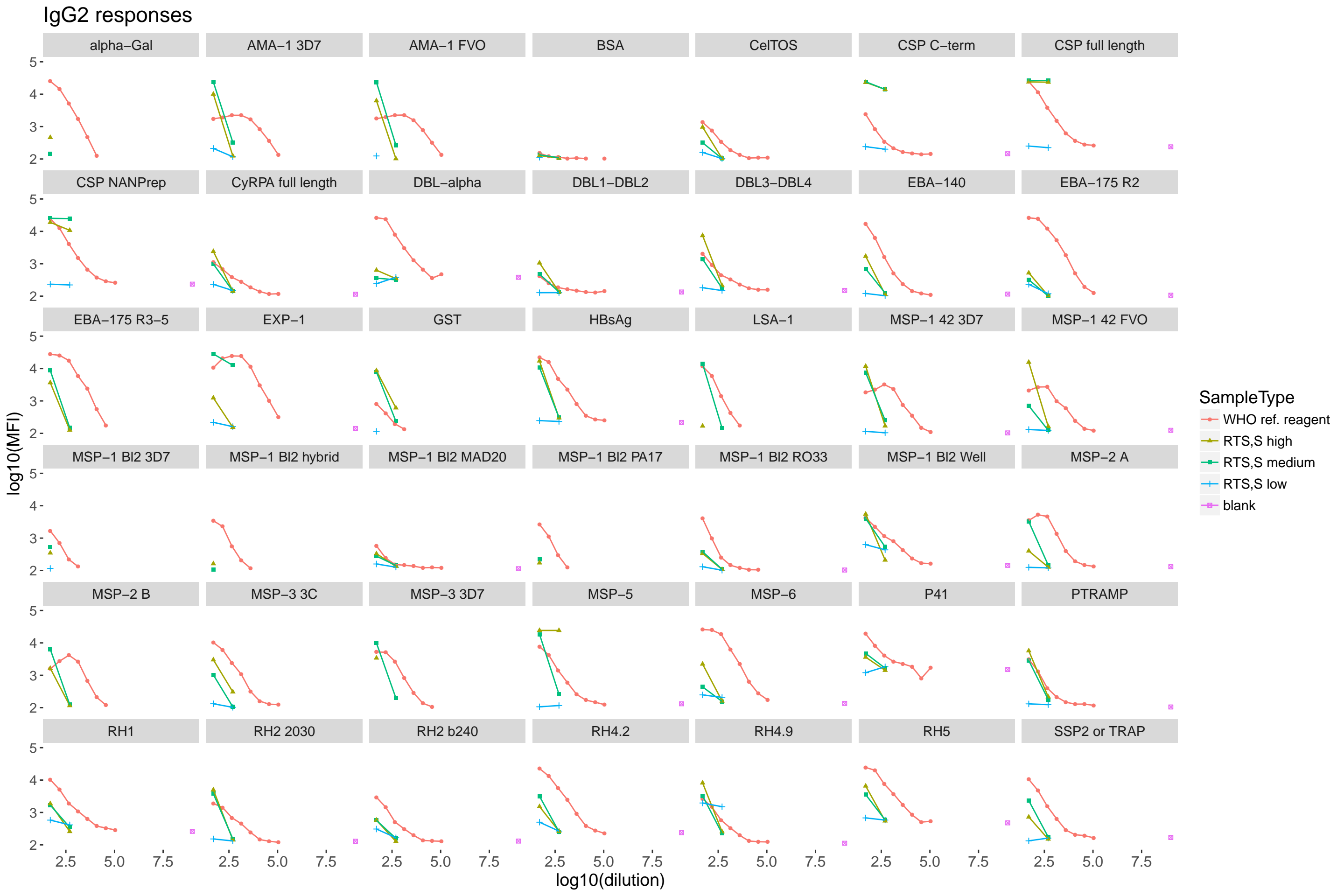

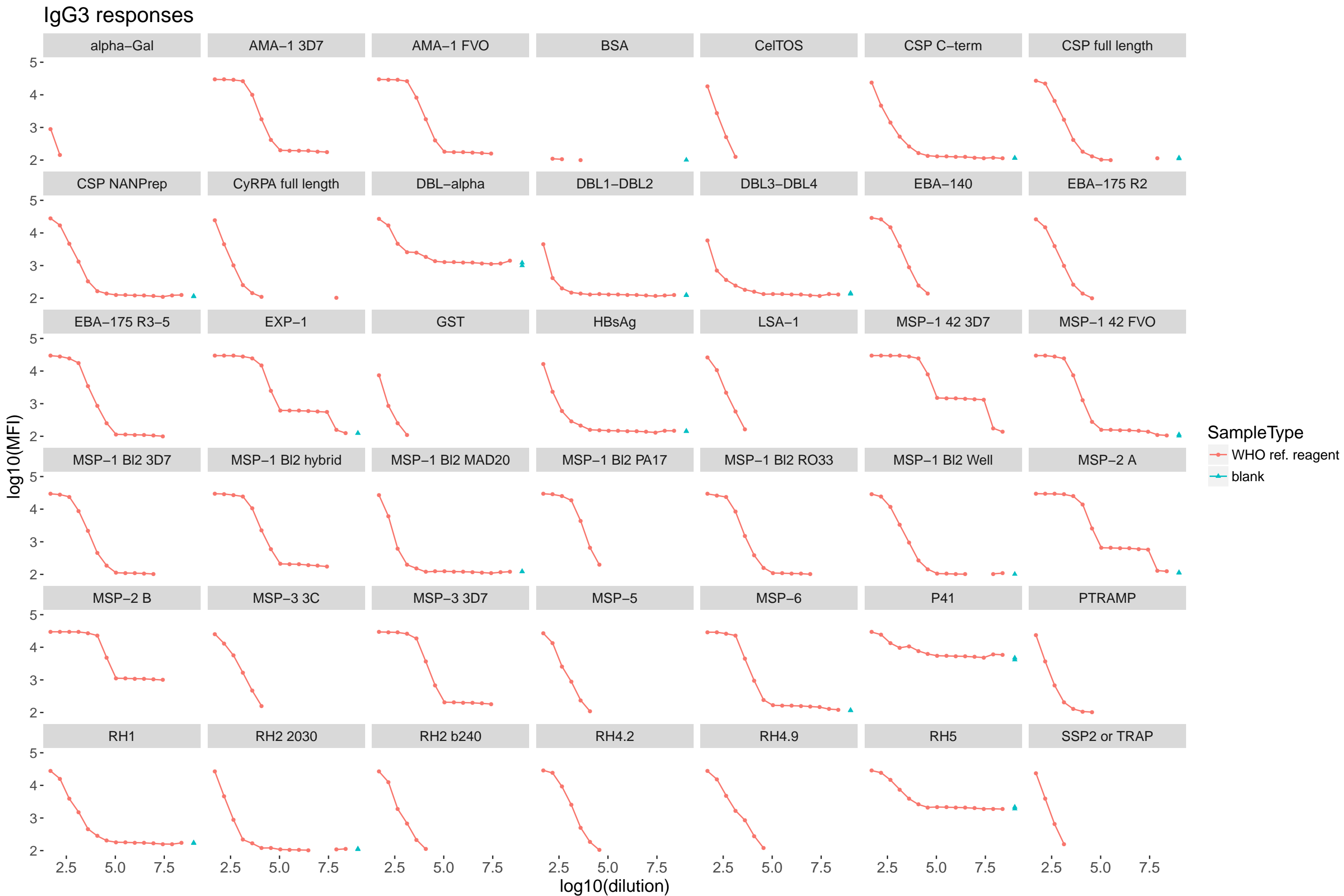

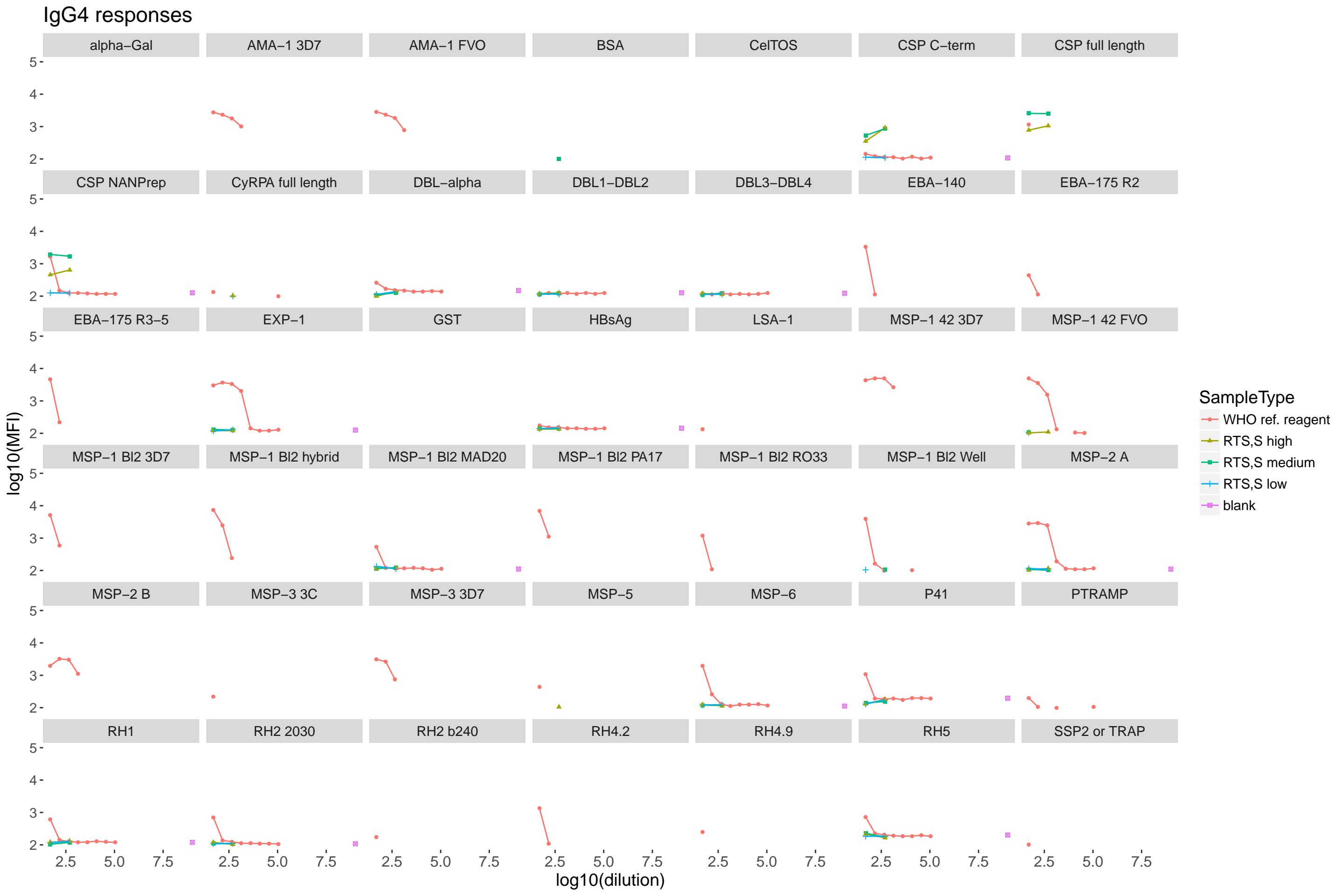

# IgM responses

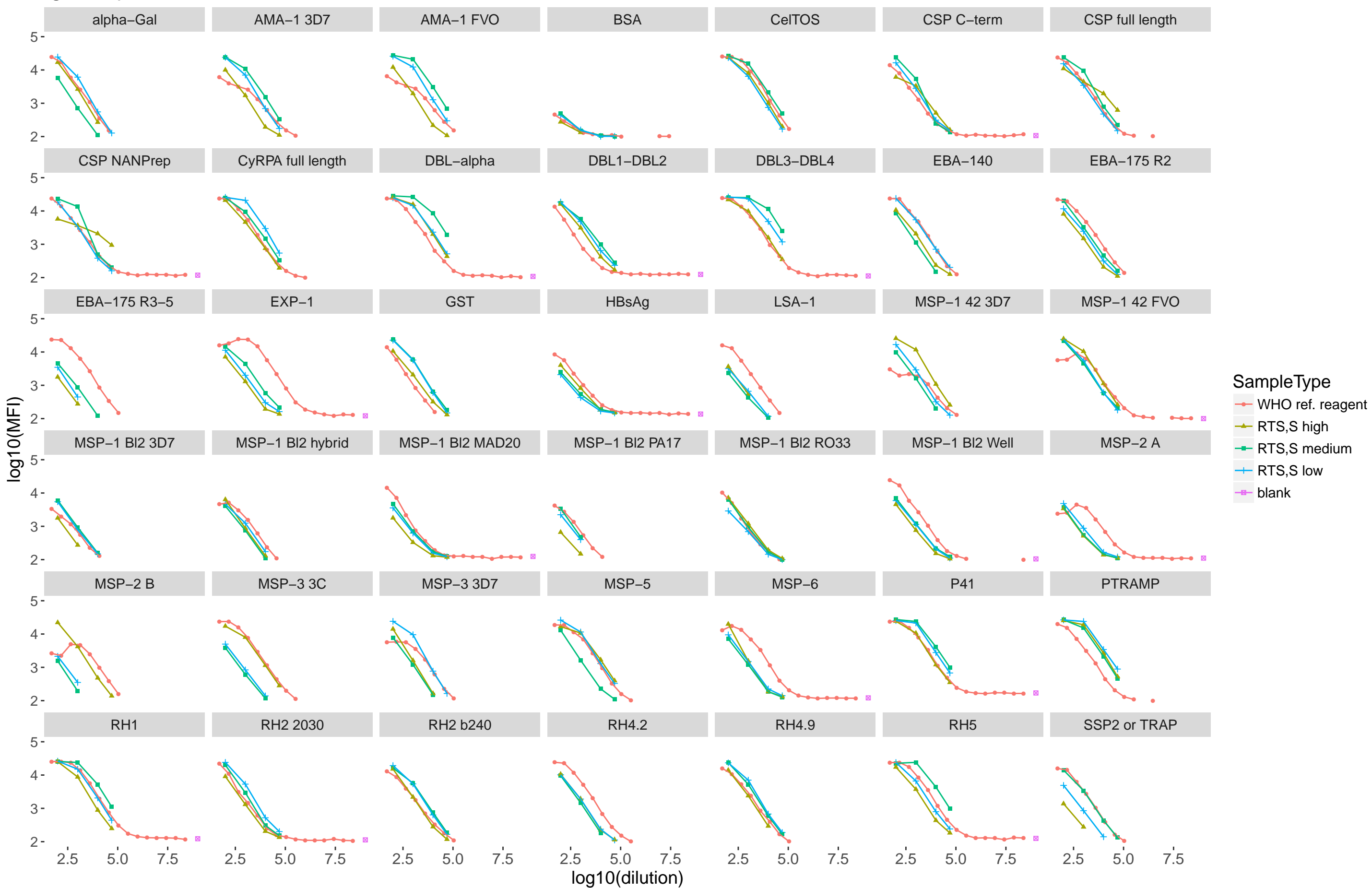

# IgM pool IgM responses

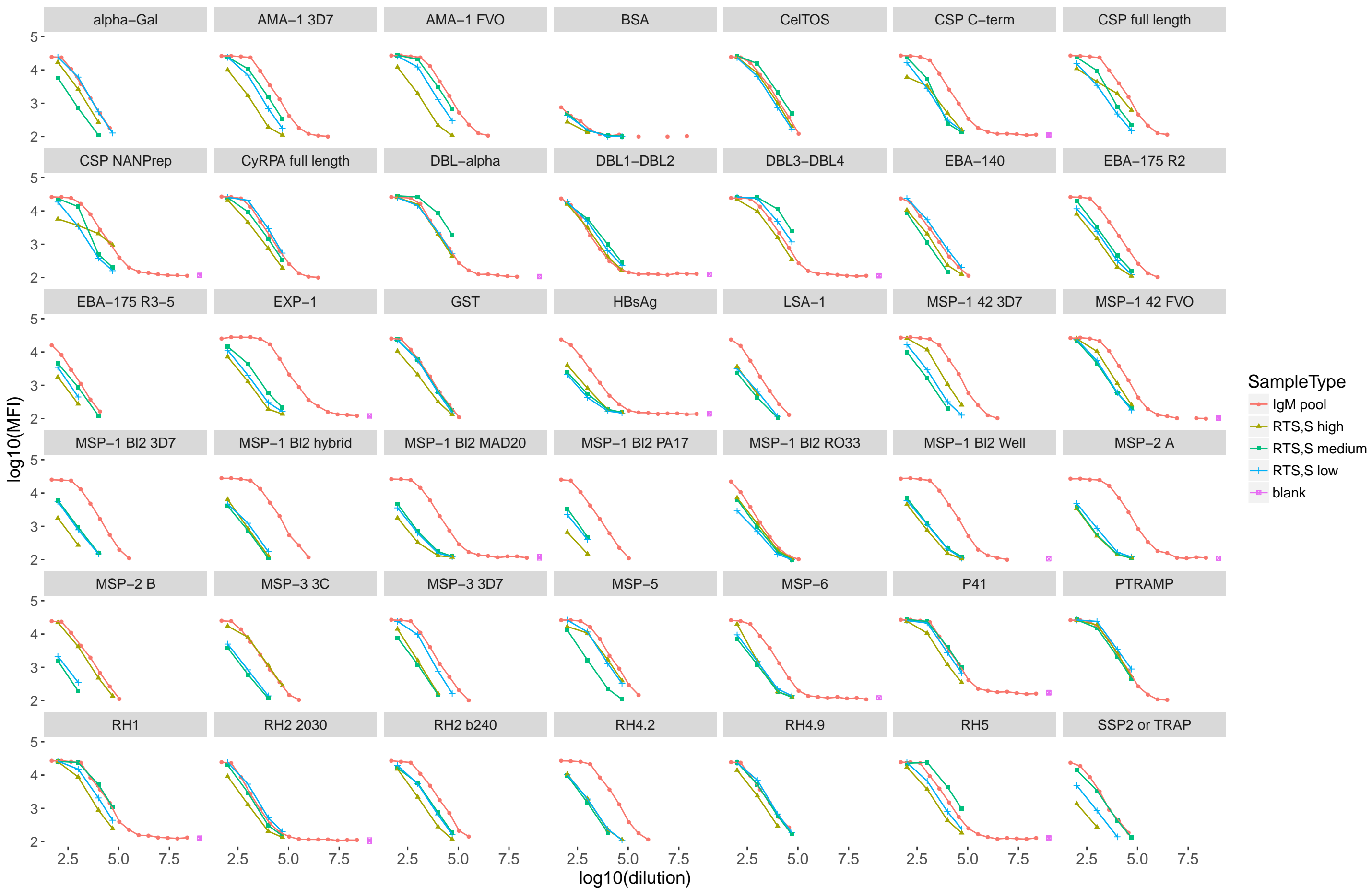

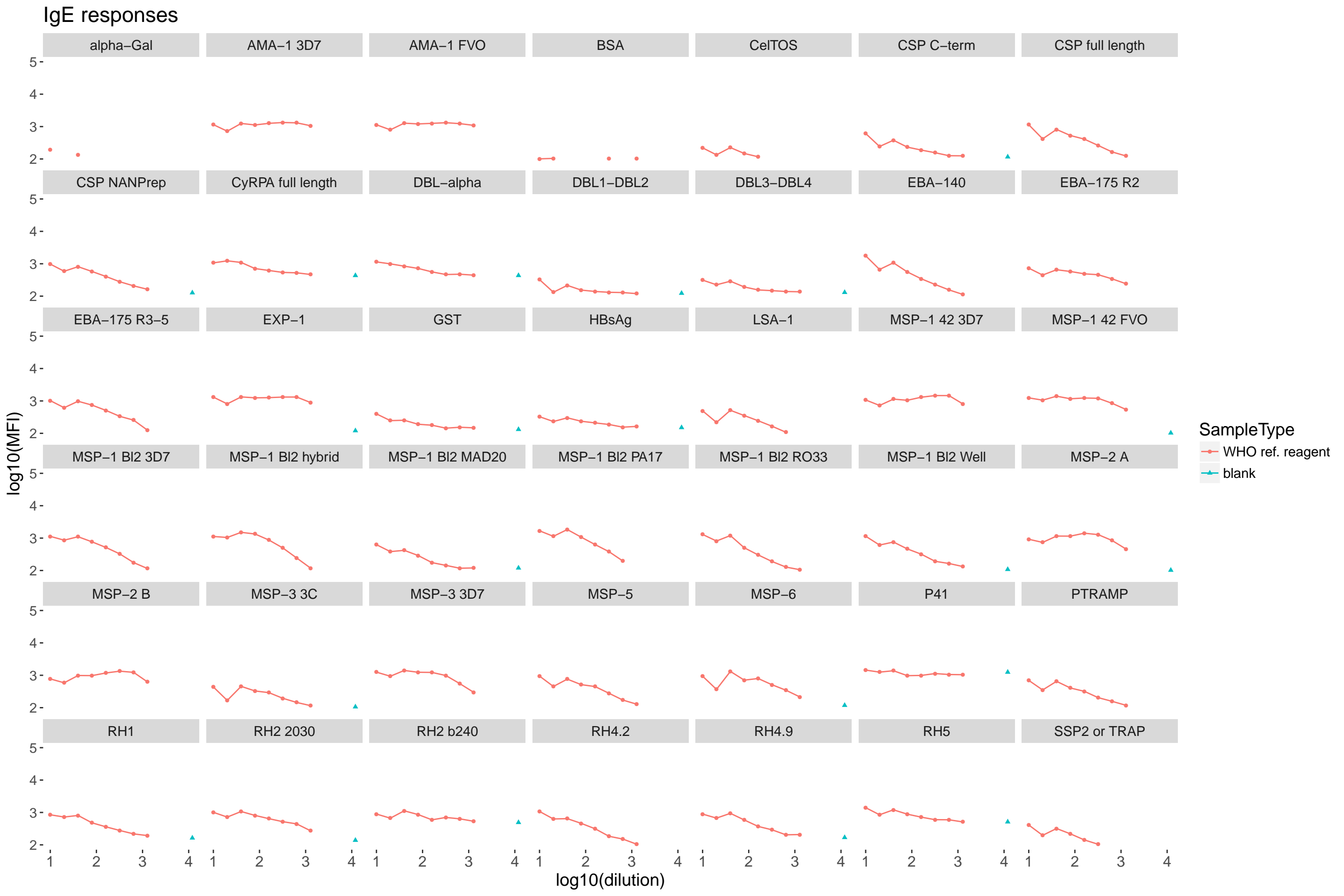

Supplement: Supplementary file 2 — Additional file 2. Comparison of the WHO reference reagent, IgM pool and RTS,S samples responses to the 40-antigen multiplex panel incubating at 4 °C ON. IgG, IgG1–4 subclasses, IgM and IgE were measured in the respective pools and samples. The plots represent the levels of antibodies measured in serial dilutions of the positive pools (1:3 starting at 1:50 for IgG, IgG1–4 and IgM; and 1:2 starting at 1:10 for IgE), and the RTS,S samples (1:10 starting at 1:500 for IgG, 1:100 for IgM, 1:50 for IgG1–4; and 1:2 starting at 1:10 for IgE). Data on IgG3 and IgE levels measured in RTS,S vaccinees were not available. Isolated dots represent the levels measured in the technical blanks. [file 12936_2018_2369_MOESM2_ESM.pdf]

IgG fitted

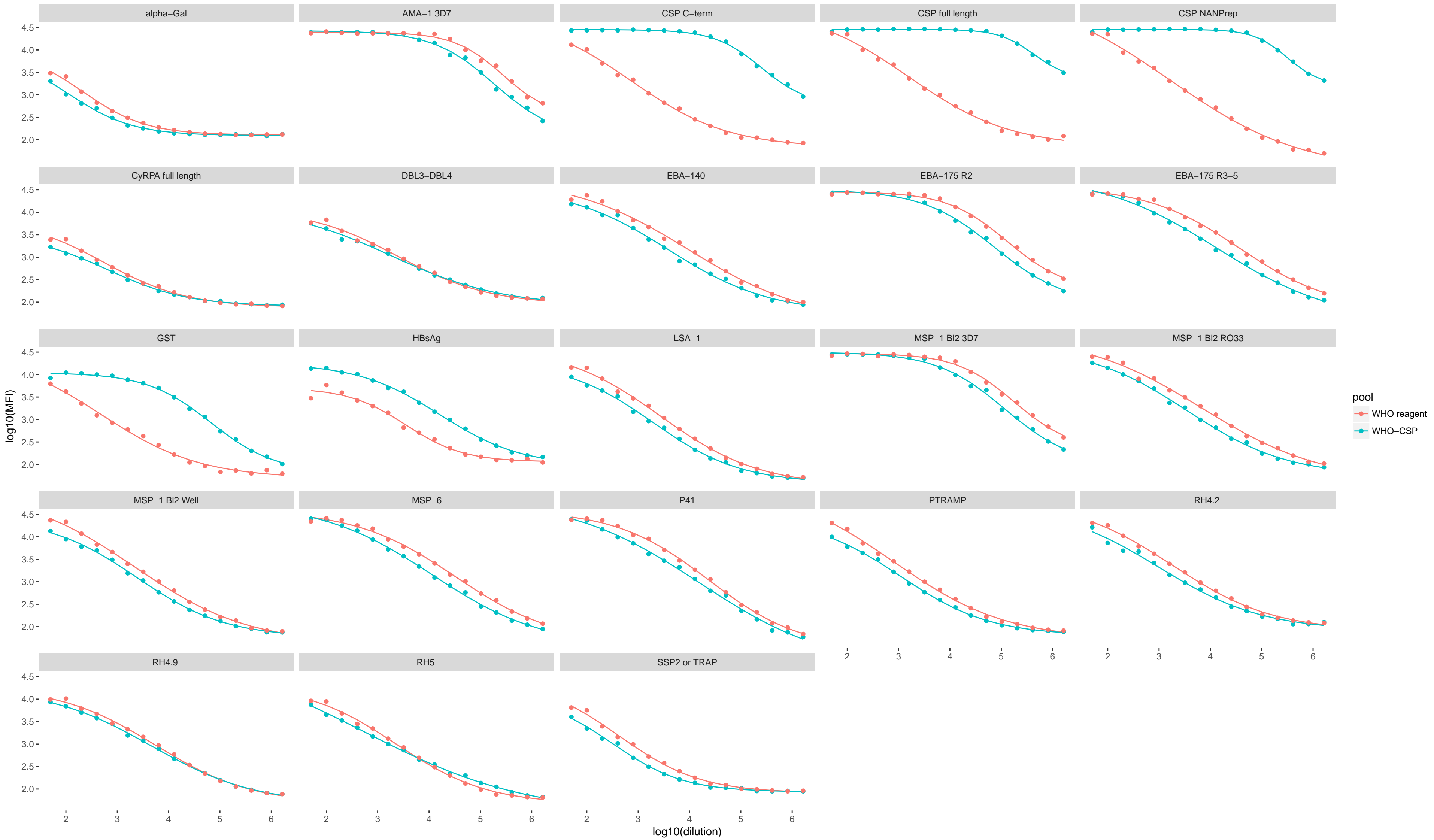

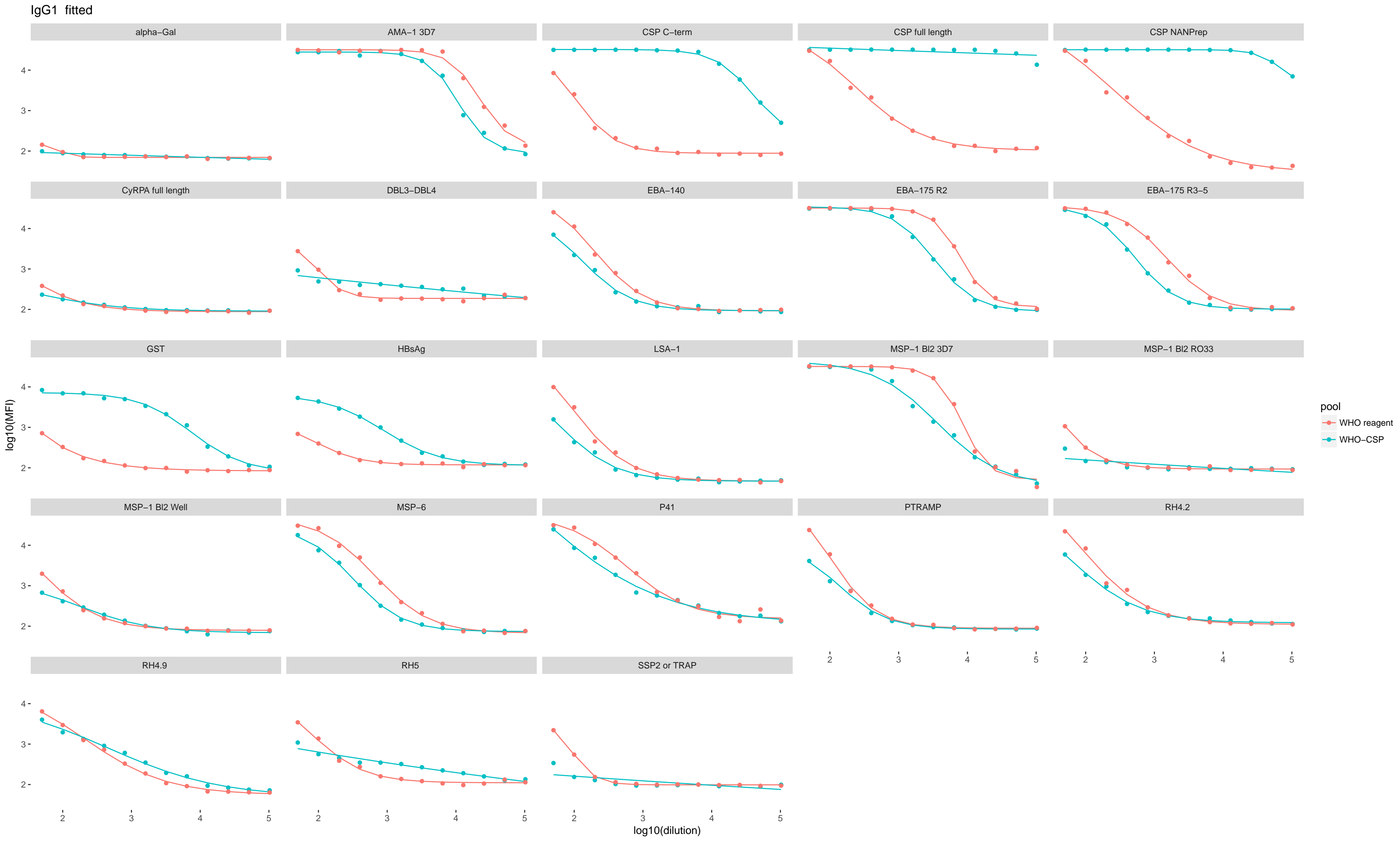

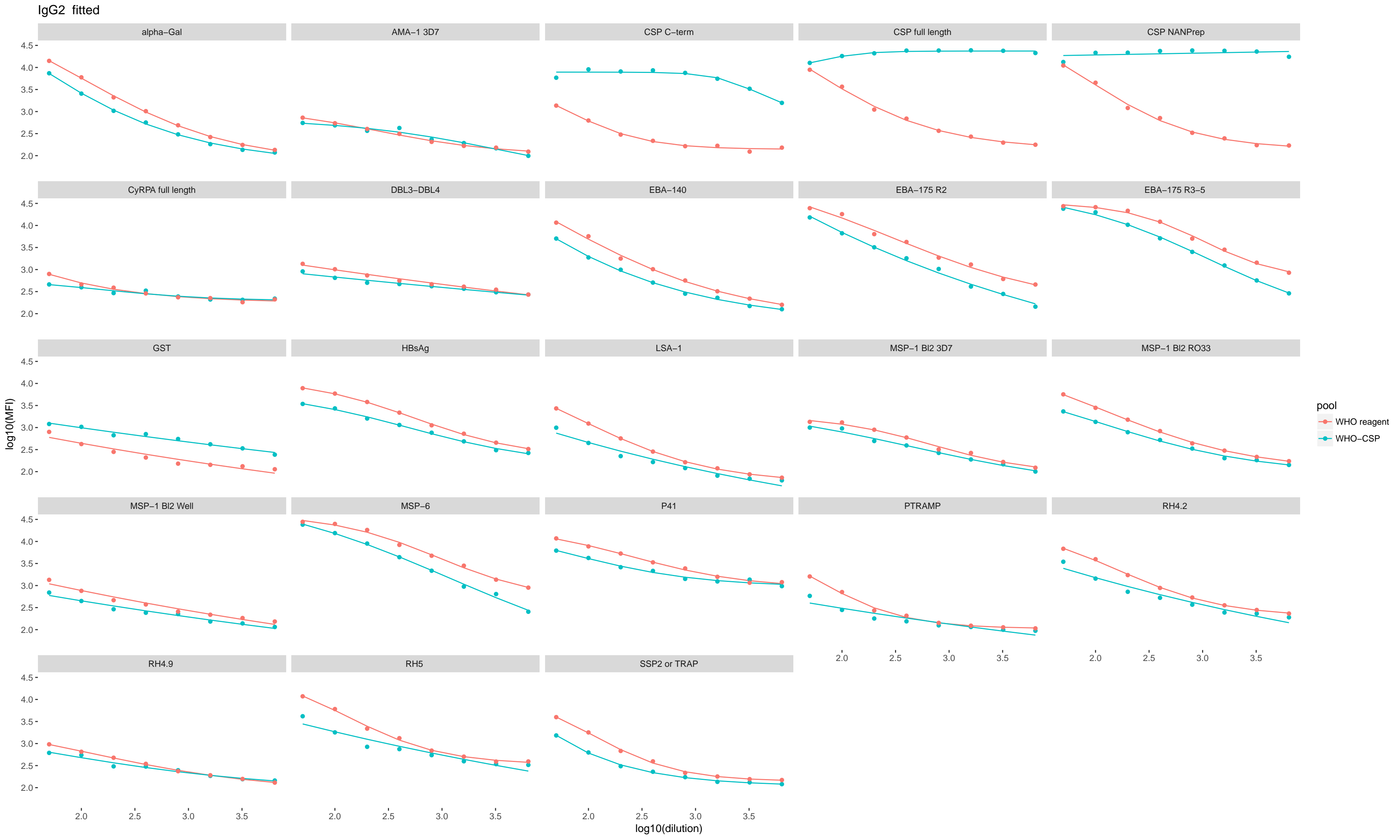

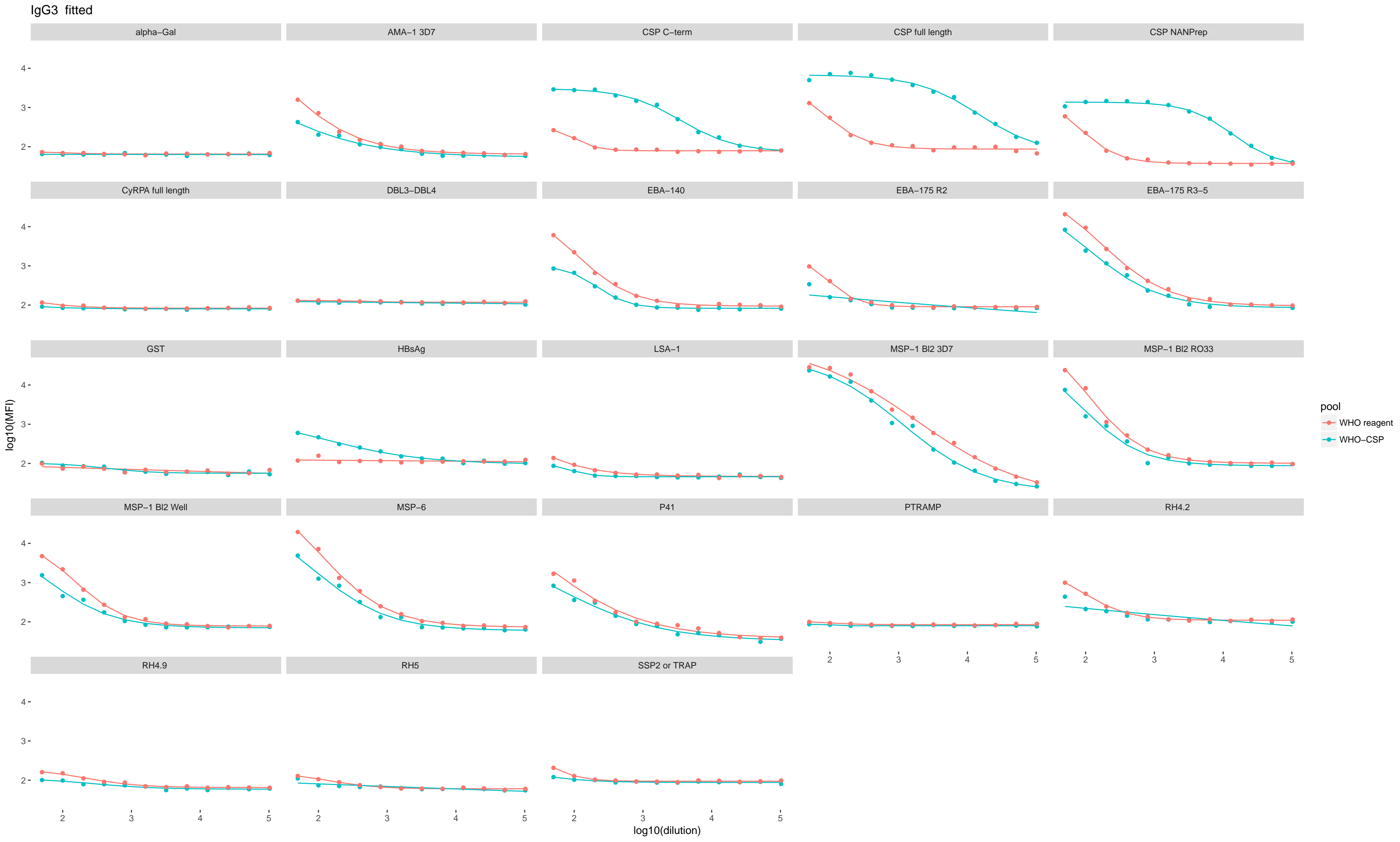

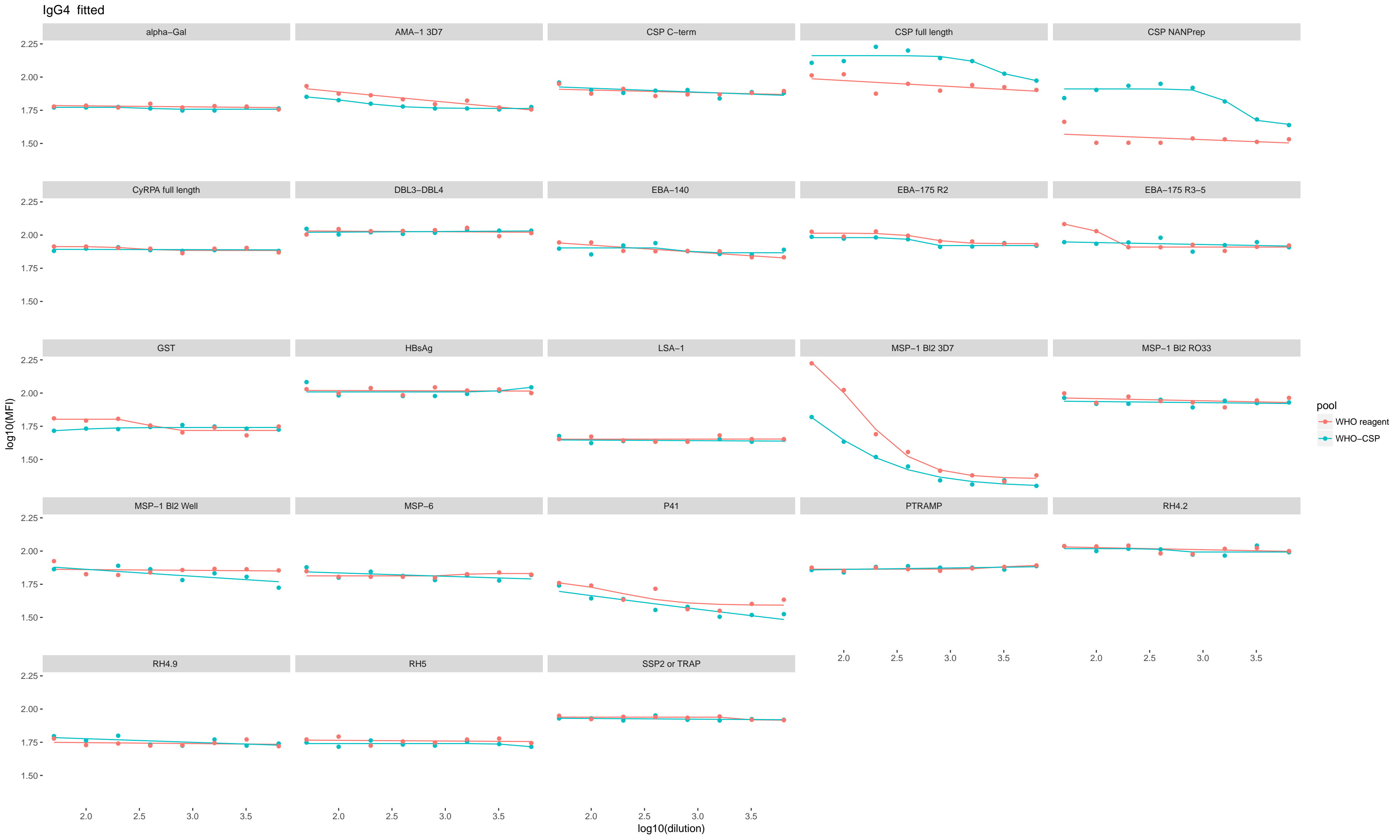

Supplement: Supplementary file 3 — Additional file 3. Comparison of the IgG and IgG1–4 predicted curves between the WHO reference reagent and the WHO-CSP pool incubating at 4 °C ON. IgG and IgG1–4 predicted curves from a non-linear equation were measured against a 23-multiplex panel. Isolated dots represent the levels measured in the technical blanks. [file 12936_2018_2369_MOESM3_ESM.pdf]

RTS,S vaccinees (n=129)

### RTS,S antigens

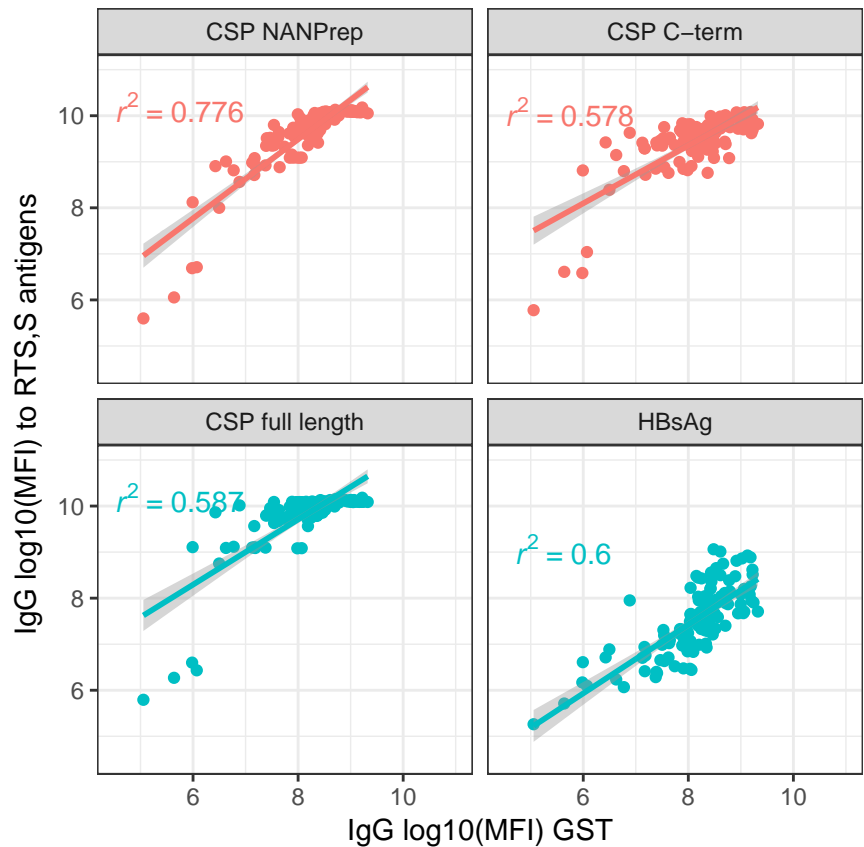

GST-fused  
 not GST-fused

### Non-RTS,S antigens

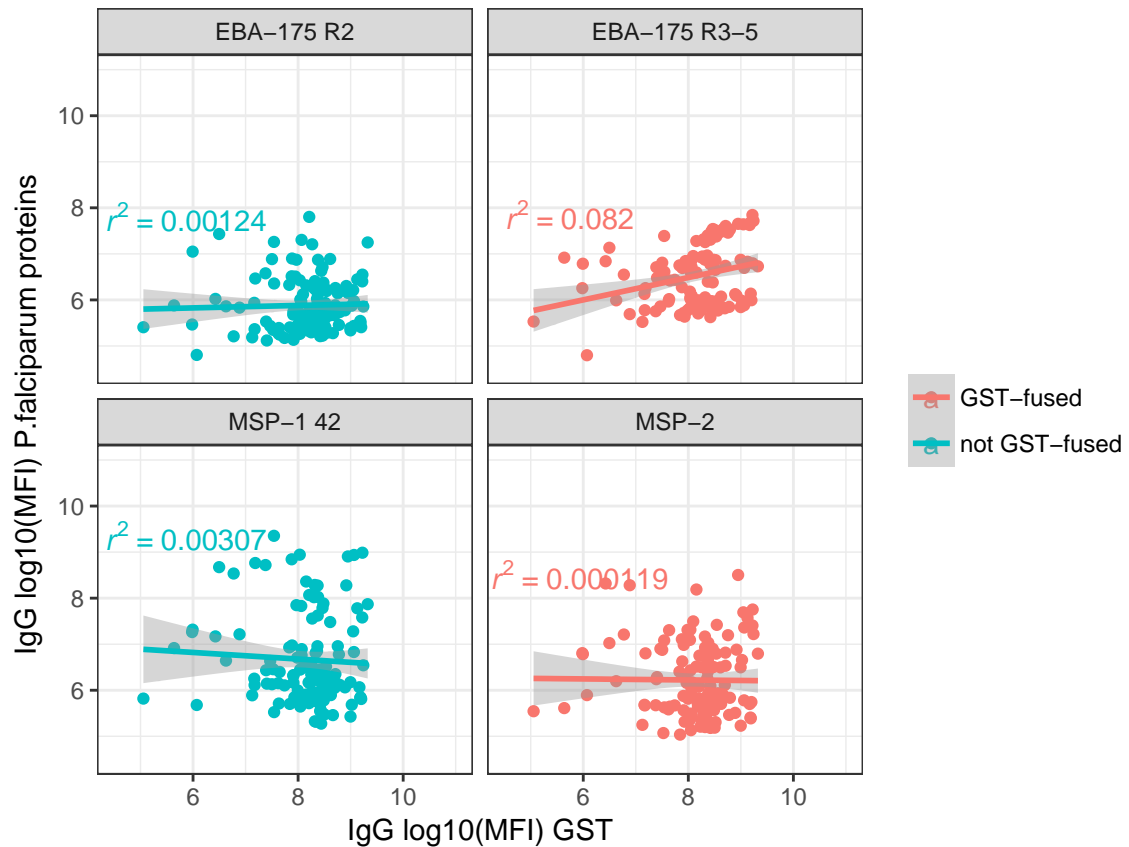

GST-fused  
 not GST-fused

Supplement: Supplementary file 5 — Additional file 5. Correlations between GST vs. antigens included in the RTS,S vaccine, and GST vs. non-RTS,S antigens in plasmas from RTS,S-vaccinated children. Scatterplots with levels of IgG (log10MFI) to GST alone in the X-axis and to GST-fused proteins (orange) or proteins not fused to GST (green) in the Y-axis. Linear regression lines with 95% confidence intervals (in grey) and Spearman correlation coefficients (r2) for each antigen. Correlations between IgGs to RTS,S proteins and GST were high but similar between GST-fused (CSP NANP & C-terminus) and non GST-fused proteins (CSP full length and HBsAg). Antibody levels against the GST-fused CSPs (Y-axis value) were higher than to the GST alone (X-axis value). IgG levels to GST fusion proteins representing non-RTS,S antigens (e.g. EBA-175, MSP-2) were not correlated with IgG levels to GST alone. There were low antibody responses to these antigens while there was a higher signal to the GST alone. Overall, the patterns of correlations were similar between GST-fused and non-GST fused proteins. Responses to GST and to GST fusion proteins appeared to be independent. [file 12936_2018_2369_MOESM5_ESM.pdf]

## IgG

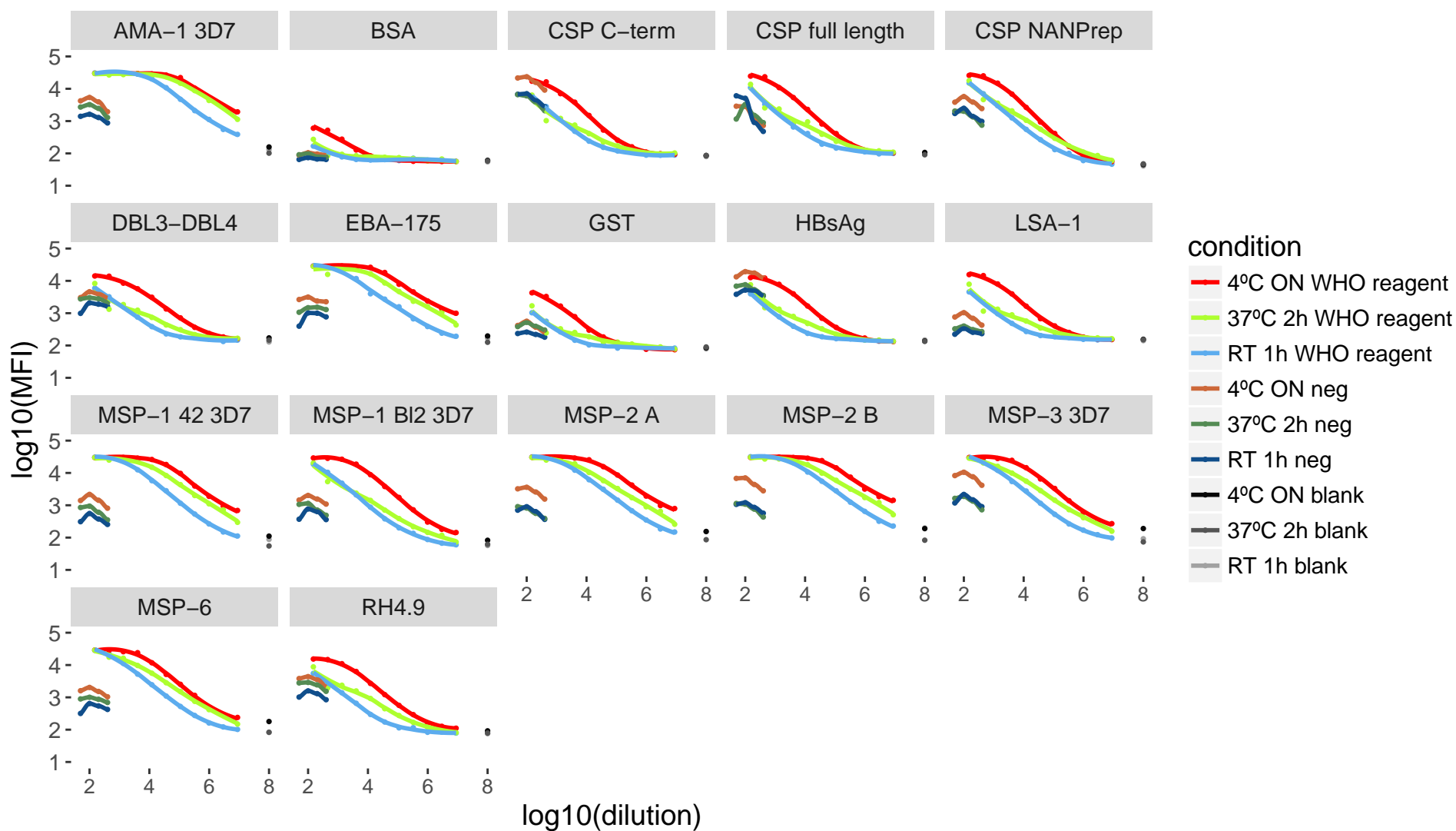

## IgG3

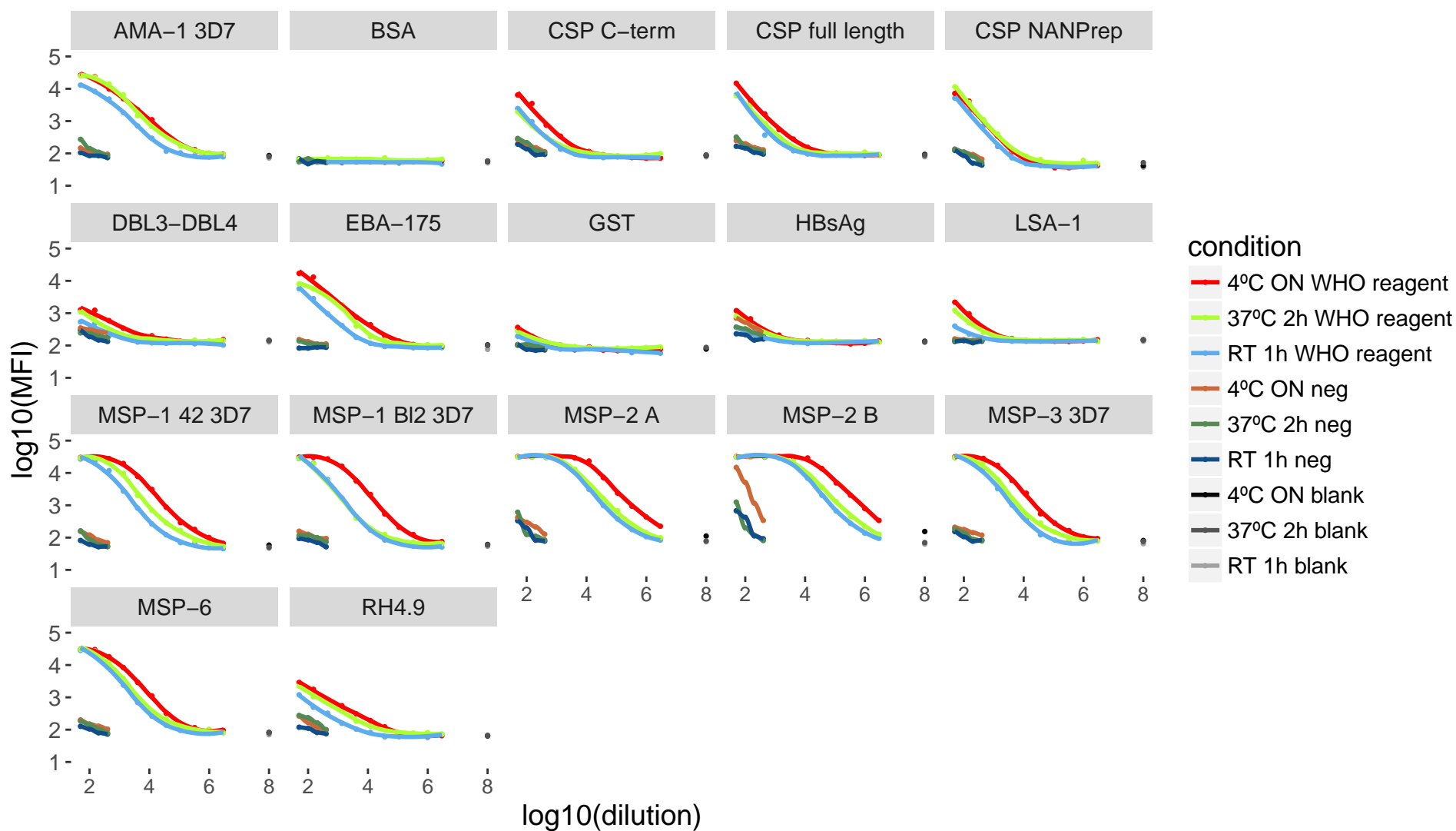

## IgG2

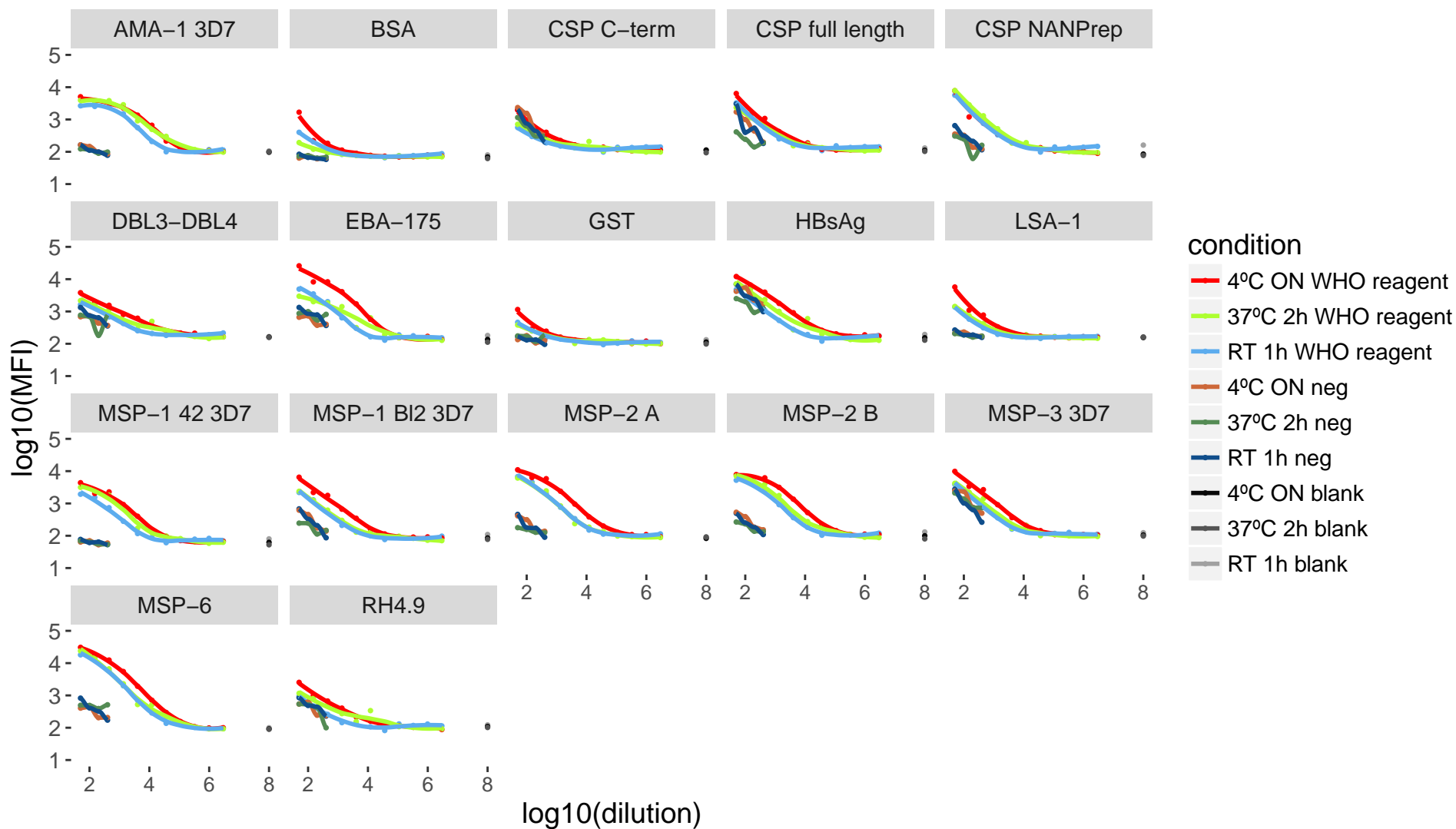

## IgG4

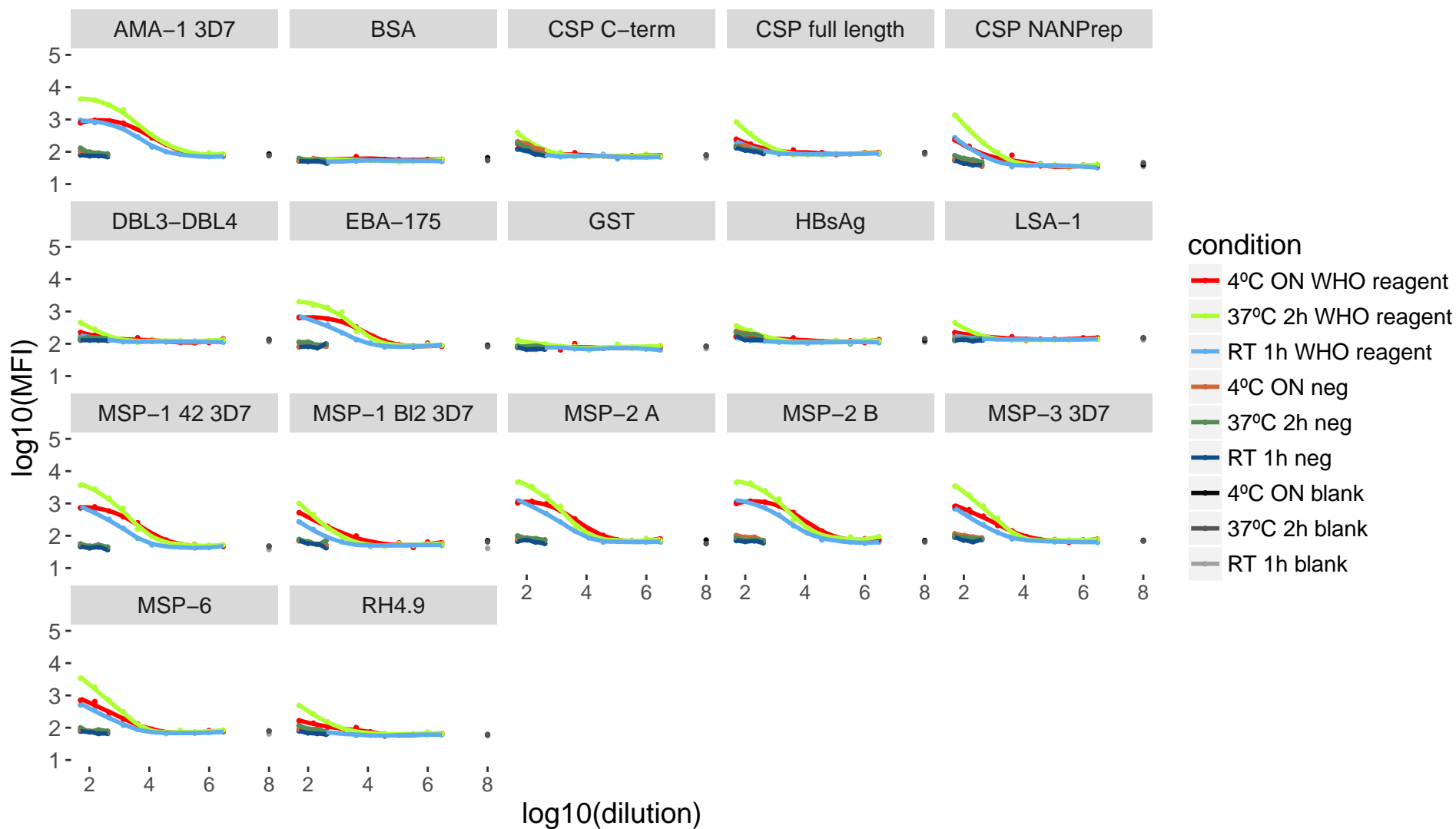

Supplement: Supplementary file 6 — Additional file 6. Levels of IgG and IgG2-4 to 15 antigens measured in the WHO reference reagent compared to negative control and blanks under three different incubation conditions. Curve plots of the antigen-specific antibody levels measured in serial dilutions of the WHO reference reagent, negative control and blanks at three different incubation conditions: 37 °C 2 h (37 °C 2 h), 4 °C overnight (4 °C ON) and room temperature for 1 h (RT 1 h). “neg” means negative control. [file 12936_2018_2369_MOESM6_ESM.pdf]
