# Supplementary material for: Optimization of incubation conditions of Plasmodium falciparum antibody multiplex assays to measure IgG, IgG1–4, IgM and IgE using standard and customized reference pools for sero-epidemiological and vaccine studies
Source: Malar J. 2018 Jun 1;17:219. doi: 10.1186/s12936-018-2369-3 (PMC5984756; doi:10.1186/s12936-018-2369-3)
Supplement: Supplementary file 4 — Additional file 4. IgG and IgG1–4 50% effective concentrations (EC50) to RTS,S-specific antigens measured in the WHO reference reagent and the WHO-CSP pool, and EC50 ratios between pools. The functions used to fit the standard curves were 4PL (SSl4) or exponential (SSexp) equations. [file 12936_2018_2369_MOESM4_ESM.pdf]

		WHO Reference reagent pool			WHO-CSP pool			
IgG type	Analyte	EC50	EC50 Std Error	Function	EC50	EC50 Std Error	Function	RATIO (EC50 $_{WHO}$/ EC50 $_{WHO-CSP}$)
IgG	CSP C-term	2.863	0.147	SSl4	5.398	0.062	SSl4	0.530
IgG	CSP full length	3.197	0.150	SSl4	5.636	0.063	SSl4	0.567
IgG	CSP NANPrep	3.235	0.201	SSl4	5.499	0.034	SSl4	0.588
IgG	GST	2.693	0.260	SSl4	4.824	0.057	SSl4	0.558
IgG	HBsAg	3.547	0.089	SSl4	4.209	0.040	SSl4	0.843
IgG1	CSP C-term	2.023	0.095	SSl4	4.597	0.032	SSl4	0.440
IgG1	CSP full length	2.393	0.114	SSl4	NA	NA	SSexp	
IgG1	CSP NANPrep	2.407	0.261	SSl4	4.835	0.015	SSl4	0.498
IgG1	GST	0.188	3.477	SSl4	3.877	0.048	SSl4	0.049
IgG1	HBsAg	1.977	0.140	SSl4	2.945	0.028	SSl4	0.671
IgG2	CSP C-term	1.806	0.358	SSl4	3.565	0.254	SSl4	0.507
IgG2	CSP full length	1.723	0.505	SSl4	1.789	0.543	SSl4	0.963
IgG2	CSP NANPrep	1.971	0.274	SSl4	NA	NA	SSexp	
IgG2	GST	NA	NA	SSexp	NA	NA	SSexp	
IgG2	HBsAg	2.746	0.035	SSl4	2.694	0.116	SSl4	1.019
IgG3	CSP C-term	2.020	0.050	SSl4	3.548	0.039	SSl4	0.569
IgG3	CSP full length	1.949	0.193	SSl4	4.166	0.105	SSl4	0.468
IgG3	CSP NANPrep	1.947	0.069	SSl4	4.141	0.048	SSl4	0.470
IgG3	GST	NA	NA	SSexp	2.632	0.217	SSl4	
IgG3	HBsAg	NA	NA	SSexp	2.085	0.424	SSl4	
IgG4	CSP C-term	NA	NA	SSexp	NA	NA	SSexp	
IgG4	CSP full length	NA	NA	SSexp	3.393	0.222	SSl4	
IgG4	CSP NANPrep	NA	NA	SSexp	3.284	0.104	SSl4	
IgG4	GST	2.599	25526.704	SSl4	1.787	3.217	SSl4	1.454
IgG4	HBsAg	NA	NA	SSexp	3.526	107889.523	SSl4	
