# Supplementary material for: Optimization of incubation conditions of Plasmodium falciparum antibody multiplex assays to measure IgG, IgG1–4, IgM and IgE using standard and customized reference pools for sero-epidemiological and vaccine studies
Source: Malar J. 2018 Jun 1;17:219. doi: 10.1186/s12936-018-2369-3 (PMC5984756; doi:10.1186/s12936-018-2369-3)

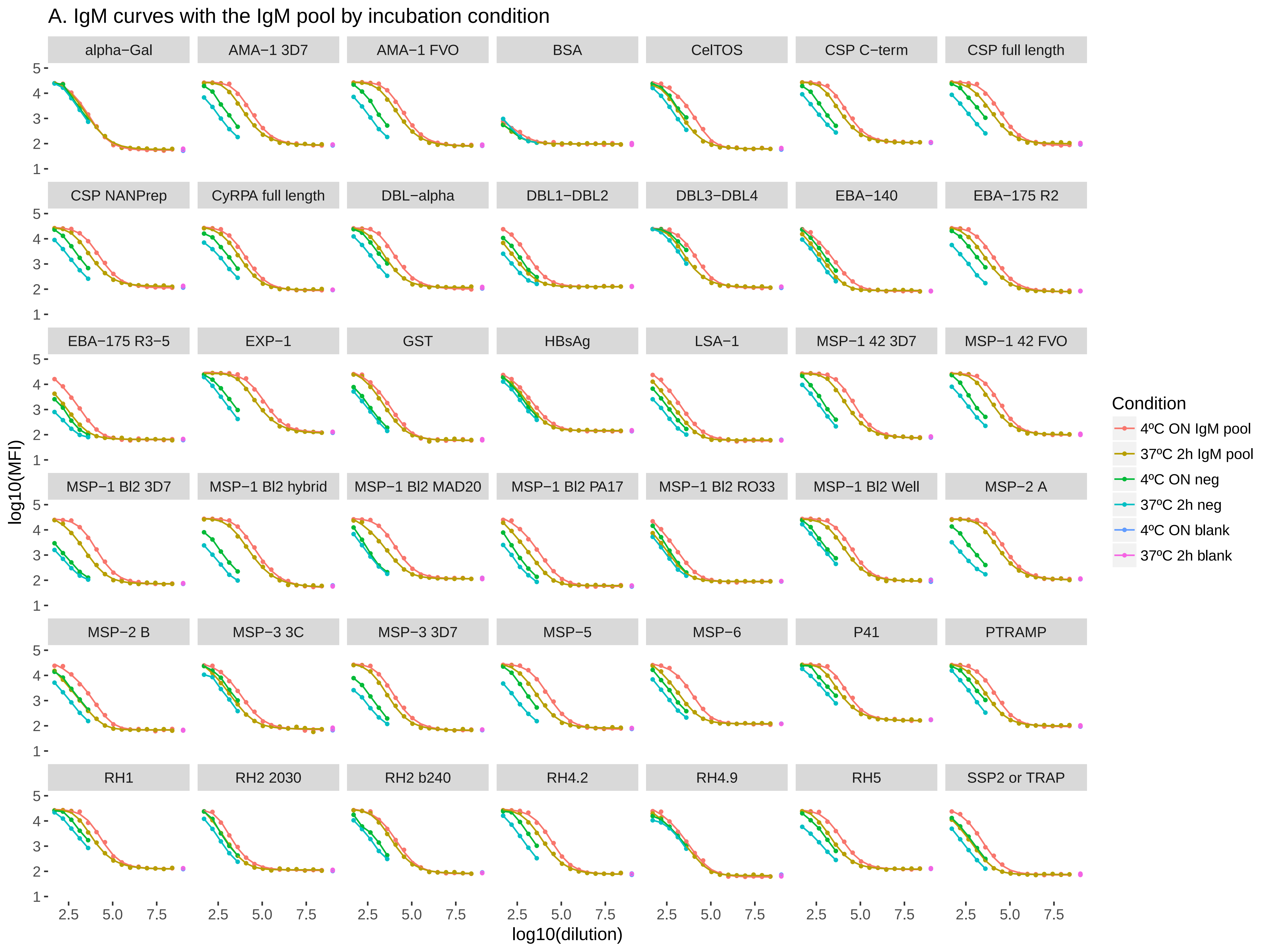
A. IgM curves with the IgM pool by incubation condition
alpha−Gal
AMA−1 3D7
AMA−1 FVO
BSA
CelTOS
CSP C−term
CSP full length
CSP NANPrep
CyRPA full length
DBL−alpha
DBL1−DBL2
DBL3−DBL4
EBA−140
EBA−175 R2
EBA−175 R3−5
EXP−1
GST
HBsAg
LSA−1
MSP−1 42 3D7
MSP−1 42 FVO
MSP−1 Bl2 3D7
MSP−1 Bl2 hybrid
MSP−1 Bl2 MAD20
MSP−1 Bl2 PA17
MSP−1 Bl2 RO33
MSP−1 Bl2 Well
MSP−2 A
MSP−2 B
MSP−3 3C
MSP−3 3D7
MSP−5
MSP−6
P41
PTRAMP
RH1
RH2 2030
RH2 b240
RH4.2
RH4.9
RH5
SSP2 or TRAP
log10(MFI)
log10(dilution)
Condition
4ºC ON IgM pool
37ºC 2h IgM pool
4ºC ON neg
37ºC 2h neg
4ºC ON blank
37ºC 2h blank

B. IgM curves with the WHO reference reagent by incubation condition

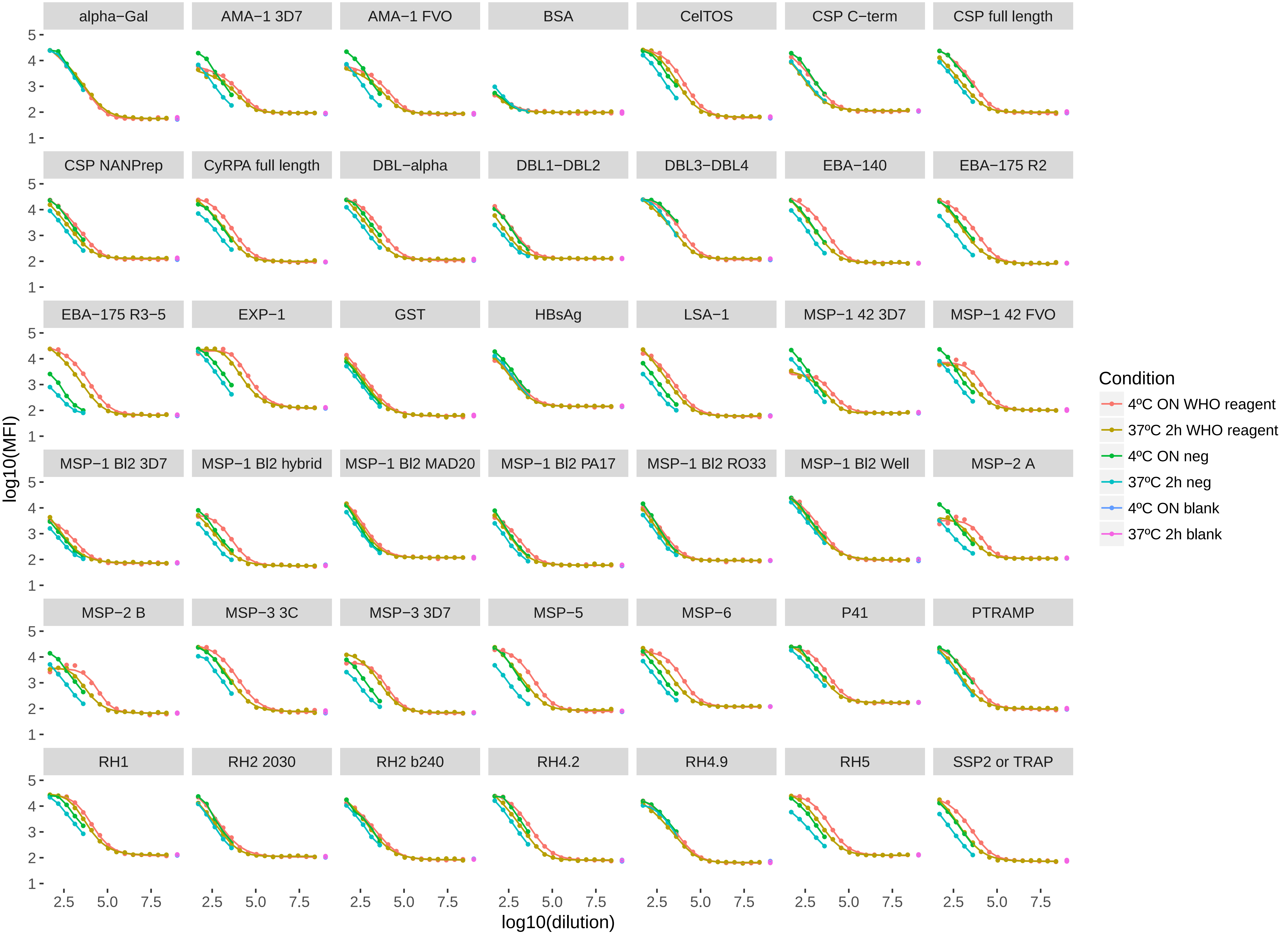

C. IgE curves with the WHO reference reagent by incubation condition

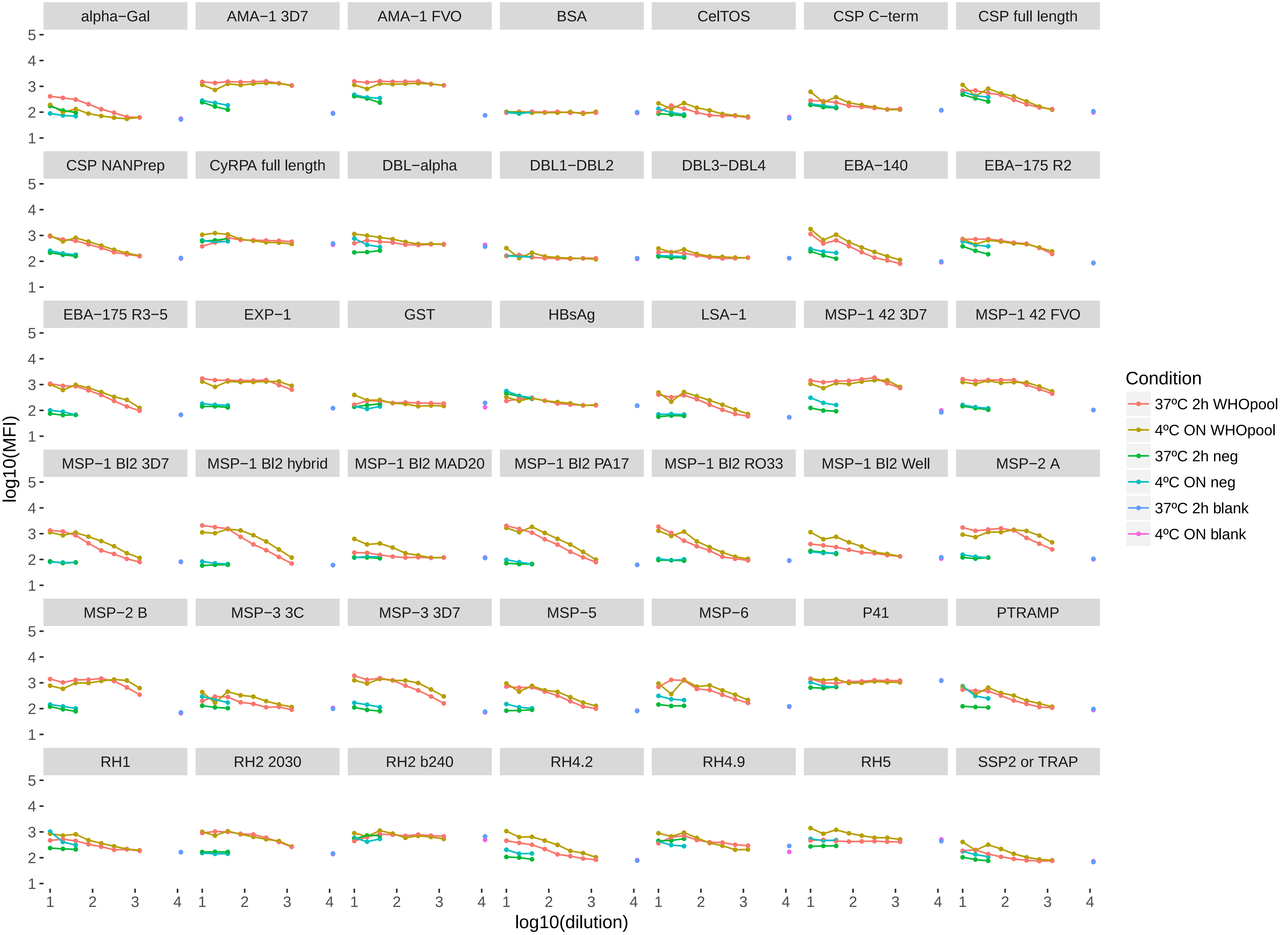

Supplement: Supplementary file 7 — Additional file 7. Levels of IgM and IgE measured to the 40-multiplex panel in the WHO reference reagent and IgM pool compared to negative control and blanks under two different incubation conditions. Incubation conditions compared are: 4 °C (4 °C ON) vs 2 h at 37 °C (37 °C 2 h). A) Predicted 5PL curves of IgM levels in the IgM pool. B) Predicted 5PL curves of IgM levels in the WHO reference reagent. C) IgE levels in the WHO reference reagent. [file 12936_2018_2369_MOESM7_ESM.pdf]
